# Walking and subjective sleep quality in adults: a Bayesian three-level meta-analysis with probabilistic clinical relevance assessment and dose–response modeling

**DOI:** 10.3389/fpubh.2026.1864607

**Published:** 2026-06-15

**Authors:** Xiangyu Kong, Xiaoying Wang, Ziwei Li, Xuhui Li

**Affiliations:** 1 School of Physical Education, Liaocheng University, Liaocheng, Shandong, China; 2 Gongjiadao Primary School, Yantai, Shandong, China; 3 The Second Middle School of Hohhot, Hohhot, Inner Mongolia, China; 4 School of Physical Education, Inner Mongolia Normal University, Hohhot, Inner Mongolia, China

**Keywords:** clinical relevance, dose–response, meta-analysis, sleep, walking

## Abstract

**Objective:**

To evaluate the effect of walking on subjective sleep quality in adults using a Bayesian three-level meta-analysis and to identify moderators of treatment response.

**Methods:**

PubMed, Embase, the Cochrane Library, and Web of Science were searched from inception through March 2026 for randomized controlled trials (RCTs) comparing walking interventions with inactive or non-aerobic controls. Effect sizes were calculated as Hedges’ g. A Bayesian three-level random-effects model was fitted to handle dependent effect sizes. Clinical relevance was assessed using posterior probabilities of exceeding the minimal clinically important difference (MCID, defined as g ≤ −0.39). Moderators were examined through subgroup analyses and meta-regressions.

**Results:**

Twenty-one RCTs (1,707 participants, 66 effect sizes) were included. Walking produced a medium-to-large improvement in subjective sleep quality (g = −0.76, 95% credible interval: −0.99 to −0.55), with extreme Bayesian evidence (Bayes factor > 10^5^) and a 99.97% posterior probability of exceeding the MCID. The 95% prediction interval fell entirely below zero. Baseline Pittsburgh Sleep Quality Index score was the strongest moderator (*β* = −0.302), with worse baseline sleep associated with larger effects. Supervision mode and exercise setting did not meaningfully influence outcomes. GRADE certainty of evidence was very low.

**Conclusion:**

Current evidence suggests that walking may improve subjective sleep quality in adults, with a 99.97% posterior probability of reaching the prespecified minimal clinically important difference, although evidence certainty is very low. Moderate-intensity walking three to five times weekly, 30 to 60 min per session, for 12 to 24 weeks may serve as a feasible prescription window, particularly for those with poorer baseline sleep. Future RCTs incorporating objective sleep measures are needed.

**Systematic review registration:**

PROSPERO, CRD420261351897.

## 1 Introduction

Insomnia and related sleep disturbances represent a major global public health concern (1). A recent systematic review estimated that 16.2% of adults worldwide experience insomnia, with 7.9% meeting criteria for severe insomnia (1). Insomnia impairs daytime functioning and quality of life, and is associated with increased risks of cardiovascular disease, metabolic disorders, and mental health conditions (1). The American Heart Association has incorporated sleep health as a core component of cardiovascular health assessment (2). Cognitive behavioral therapy for insomnia (CBT-I) is recommended as the first-line non-pharmacological treatment for chronic insomnia in adults (3), yet its dissemination remains limited by a shortage of trained therapists, substantial time requirements, and variable patient adherence (4). Exercise interventions consistently improve subjective sleep quality (5–7) and represent a viable approach for addressing this accessibility gap.

However, existing evidence has largely pooled heterogeneous modalities, including yoga, tai chi, resistance training, and aerobic exercise (7–9), generating substantial heterogeneity and limiting modality-specific prescriptions. Walking is one of the most common forms of moderate-intensity physical activity and is consistent with the World Health Organization recommendation that adults accumulate 150–300 min of moderate-intensity aerobic activity per week (10). It has a low entry barrier and is easy to disseminate. Recent network meta-analyses have evaluated walking as an independent modality and suggested that it ranks among the most effective interventions for improving sleep. Bu et al. (11) reported that walking or jogging yielded the largest reductions in insomnia severity across all exercise protocols. Xiong et al. (12) identified optimal and clinically meaningful threshold doses for walking in older adults. Liang et al. (5), in an analysis of 200 randomized controlled trials (RCTs), identified walking as one of the most effective exercise modalities. Even so, walking has usually appeared only as one node in network meta-analyses or absorbed into broader aerobic exercise categories. No systematic review or meta-analysis to date has examined walking alone as an intervention for sleep quality.

Methodological limitations have also persisted. Previous meta-analyses have predominantly employed frequentist frameworks (8, 9, 11), which cannot quantify evidence in favor of the null hypothesis or estimate the probability that an effect exceeds a clinical threshold. RCTs in this domain frequently report multiple intervention arms, outcome measures, or assessment time points, creating dependency structures among effect sizes that two-level models cannot adequately address. Although the minimal clinically important difference (MCID) has been applied in the exercise-sleep literature (6, 12), no study has reported posterior probabilities of exceeding the MCID. Nonlinear dose–response methods have advanced in recent years (12–14), yet systematic comparisons of the independent moderating effects and functional forms of individual walking prescription parameters remain scarce.

To address these gaps, this study employed a Bayesian three-level random-effects model to synthesize evidence on the effects of walking on subjective sleep quality in adults. Our goals were to estimate the overall effect and its clinical relevance using posterior probabilities, to identify population- and prescription-level moderators by comparing linear and nonlinear models, and to separate between-study from within-study heterogeneity, thereby informing evidence-based walking prescriptions.

## 2 Methods

### 2.1 Protocol registration

This systematic review and meta-analysis was conducted in accordance with the Preferred Reporting Items for Systematic Reviews and Meta-Analyses (PRISMA) 2020 reporting guidelines (15). The protocol was prospectively registered with PROSPERO (registration number: CRD420261351897).

### 2.2 Literature search

PubMed, Embase, the Cochrane Library, and Web of Science were searched from inception through March 2026. The search strategy was structured around three concept groups: walking exercise (Walking [MeSH], walk*, brisk walking, treadmill walking, Nordic walking), sleep (Sleep [MeSH], Sleep Wake Disorders [MeSH], sleep quality, insomnia, PSQI, ISI), and randomized controlled trials (Randomized Controlled Trial [pt], randomized, RCT). The three concept groups were combined with the Boolean operator AND. Full search strategies for each database are provided in Supplementary File 1. Reference lists of included studies were also hand-searched.

### 2.3 Eligibility criteria

Eligibility criteria were established using the population, intervention, comparison, outcome, and study design (PICOS) framework. Participants were adults aged 18 years or older, with no restrictions on health status, baseline sleep quality, or fitness level. The intervention was a structured exercise program with walking as the primary modality (general walking, brisk walking, treadmill walking, or Nordic walking) with a minimum duration of 2 weeks. Comparators were inactive or non-aerobic control conditions, including usual care, waitlist, no intervention, and health education. Stretching controls were also eligible. Stretching is a light-intensity, non-aerobic activity that imposes minimal cardiometabolic load and is commonly used as an active (attention-matched) control in exercise trials (16), so it was not treated as an aerobic exercise comparator. The primary outcome was subjective sleep quality assessed with a validated instrument. The Pittsburgh Sleep Quality Index (PSQI) global score was used preferentially. When PSQI data were unavailable, the Insomnia Severity Index (ISI) or other validated sleep scales were accepted. Only RCTs were eligible.

Studies were excluded if they used a non-RCT design, if the intervention did not employ walking as the primary component or combined walking with other treatment modalities, if the intervention lasted fewer than 2 weeks, if sleep quality outcomes were not reported, or if the publication was a duplicate report.

### 2.4 Study screening and data extraction

Two reviewers independently screened the literature. Titles and abstracts were evaluated first to exclude clearly ineligible records, followed by full-text assessment of candidate articles. Disagreements were resolved through discussion or, when necessary, adjudication by a third reviewer. Two independent reviewers extracted data using a standardized form. Extracted information included study characteristics (first author, publication year, country, funding), participant characteristics (sample size, mean age, proportion of female participants, body mass index (BMI), baseline PSQI score, population type), intervention characteristics (exercise intensity, session duration, weekly frequency, total intervention duration, supervision, exercise setting, intervention flexibility), behavior change technique (BCT) coding, and pre- and post-intervention means and standard deviations. Data presented only in graphical form were digitized using GetData Graph Digitizer (version 2.3).

Exercise intensity was expressed in metabolic equivalents (METs) (17). One MET corresponds to the standardized resting metabolic rate, conventionally defined as 3.5 mL O₂·kg^−1^·min^−1^. When original studies did not report MET values, intensity was assigned based on the walking type, speed, and conditions described, using the corresponding activity codes from the 2024 Adult Compendium of Physical Activities (18).

### 2.5 Risk of bias assessment

The Cochrane Risk of Bias 2 (RoB 2) tool (19) was used to evaluate all included studies across five domains: the randomization process, deviations from intended interventions, missing outcome data, measurement of the outcome, and selection of the reported result. Each study was independently assessed by two reviewers and rated as low risk, some concerns, or high risk. Disagreements were resolved through discussion.

### 2.6 Effect size calculation

The standardized mean difference (SMD), corrected for small-sample bias using Hedges’ g (20), served as the effect size metric. Effect sizes were computed as standardized mean change scores (21), defined as the pre-to-post mean difference divided by the pre-intervention standard deviation, multiplied by the Hedges correction factor J = 1–3/(4(*n* − 1) − 1). All scales were oriented so that higher scores indicate worse sleep, so that negative values favour walking. Because most studies did not report pre-post correlation coefficients, the primary analysis assumed r = 0.5 and used an approximate variance formula for standardized mean change scores (22). This assumption was evaluated through sensitivity analysis (see Section 2.11).

### 2.7 Statistical model

A Bayesian three-level random-effects model (23) was fitted to estimate the pooled effect size and heterogeneity for walking versus control conditions. The three levels corresponded to sampling error (known standard errors), within-study variation across multiple effect sizes from the same study (e.g., multiple walking arms, outcome measures, or time points), and between-study heterogeneity. The model was specified as smd | se(se_smd) ~ 1 + group + (1 | studyID/es_id), in which the arm-level change scores were regressed on a binary group indicator. The intercept captured the control condition and the group coefficient represented the pooled walking effect relative to control. The priors were intercept ~ Normal(0, 1), regression coefficients ~ Normal(0, 1), and random-effects standard deviations ~ Cauchy(0, 1). The model was fitted using the brms package (24) in R with the No-U-Turn Sampler (25). Eight Markov chain Monte Carlo (MCMC) chains were run for 6,000 iterations each (3,000 warmup), with adapt_delta = 0.99, yielding 24,000 post-warmup posterior samples. Convergence was assessed using the potential scale reduction factor (R^) and effective sample size (26), supplemented by posterior predictive checks (Supplementary Files 3, 6). For subgroup analyses, meta-regressions, and sensitivity analyses, models were refitted with four chains and 4,000 iterations (2,000 warmup) to balance computational efficiency and convergence.

Within the Bayesian framework, posterior means, medians, and 95% credible intervals (CrIs) are reported. The probability of direction (pd) quantified the certainty that a parameter’s posterior mass fell in a given direction (27). Bayes factors (BFs) were computed using the Savage–Dickey density ratio at the parameter level (28) and bridge sampling at the model level (29). Results were interpreted according to the classification scheme of Jeffreys (1961). The region of practical equivalence (ROPE) and highest density interval (HDI) were also computed (30) (Supplementary Files 4, 5). Heterogeneity was quantified using between-study and within-study variance components, and I^2^ was decomposed into between-study, within-study, and total components (31). The certainty of evidence for the primary outcome was appraised using the Grading of Recommendations, Assessment, Development and Evaluations (GRADE) framework (32), with potential downgrading based on risk of bias, inconsistency, indirectness, imprecision, and publication bias. Model explanatory performance was additionally summarised using Bayesian R^2^.

### 2.8 Minimal clinically important difference

The MCID threshold was adopted from the meta-analysis of 14 exercise types by Liang et al. (5) and set at Hedges’ g ≤ −0.39. That threshold was derived from 200 RCTs (*n =* 23,523) using a distribution-based approach. Adopting an externally established threshold avoids the circular dependency that arises when the same dataset is used to both define and test a threshold. Posterior probabilities of the pooled effect and individual study effects exceeding the MCID were calculated (Supplementary File 7).

### 2.9 Subgroup analysis

Subgroup analyses were performed for six categorical moderators: population type, outcome measurement instrument, overall risk of bias, supervision mode, exercise setting, and funding source. For each moderator, models with and without an interaction term were fitted and compared using Bayes factors to quantify the strength of evidence for between-subgroup differences (Supplementary File 8).

### 2.10 Meta-regression of moderators

Meta-regression analyses were conducted for 10 continuous moderators: baseline PSQI score, mean age, proportion of female participants, mean BMI, exercise intensity, total intervention duration, session duration, weekly frequency, intervention flexibility, and total number of BCTs. Because the relationship between each moderator and the effect size could be nonlinear, three competing models were fitted for each variable: a linear model, a penalized spline model with basis dimension k = 3, and a penalized spline model with basis dimension k = 4. Here k denotes the dimension of the spline basis (the number of basis functions), with larger k permitting greater flexibility, rather than the number of knots. The optimal model was selected on the basis of the approximate leave-one-out cross-validation information criterion (LOO-IC) (33). When a moderator had fewer than six unique values, only the linear model was fitted (Supplementary Files 9, 10).

### 2.11 Sensitivity analyses

Four sets of sensitivity analyses were performed to assess the robustness of the findings. Leave-one-out analysis sequentially removed each included study and refitted the primary model (Supplementary File 13). The pre-post correlation sensitivity analysis refitted the model under assumed correlation values of r = 0.3, 0.5, 0.7, and 0.9 (Supplementary File 14). The risk-of-bias sensitivity analysis compared results from the full dataset with those obtained after excluding studies rated as high risk of bias (Supplementary File 15). The prior sensitivity analysis specified five sets of priors ranging from Normal(0, 0.5) to Normal(0, 10) to examine whether posterior estimates were sensitive to prior specification (Supplementary File 12).

### 2.12 Publication bias assessment

Funnel plot asymmetry was assessed visually and tested statistically using Egger’s regression test (34) and Begg’s rank correlation test (35). The Rosenthal fail-safe N (36) was also calculated (Supplementary File 11).

### 2.13 Prediction interval and cumulative meta-analysis

A 95% prediction interval (37) was computed to estimate the range within which the true effect of a future comparable study would be expected to fall (Supplementary File 16). A cumulative meta-analysis (38) was conducted by sequentially adding studies in order of publication year to examine the stability of the pooled effect as evidence accumulated (Supplementary File 17).

### 2.14 Software

All analyses were performed in R (version 4.5.1). The brms package (24) was used to fit Bayesian multilevel models, the bayestestR package (27) to compute Bayesian inference indices, the metafor package to conduct publication bias tests, the loo package (33) for leave-one-out cross-validation, and the tidybayes package for posterior extraction and visualization.

## 3 Results

### 3.1 Study selection and characteristics of included studies

**Figure 1:**
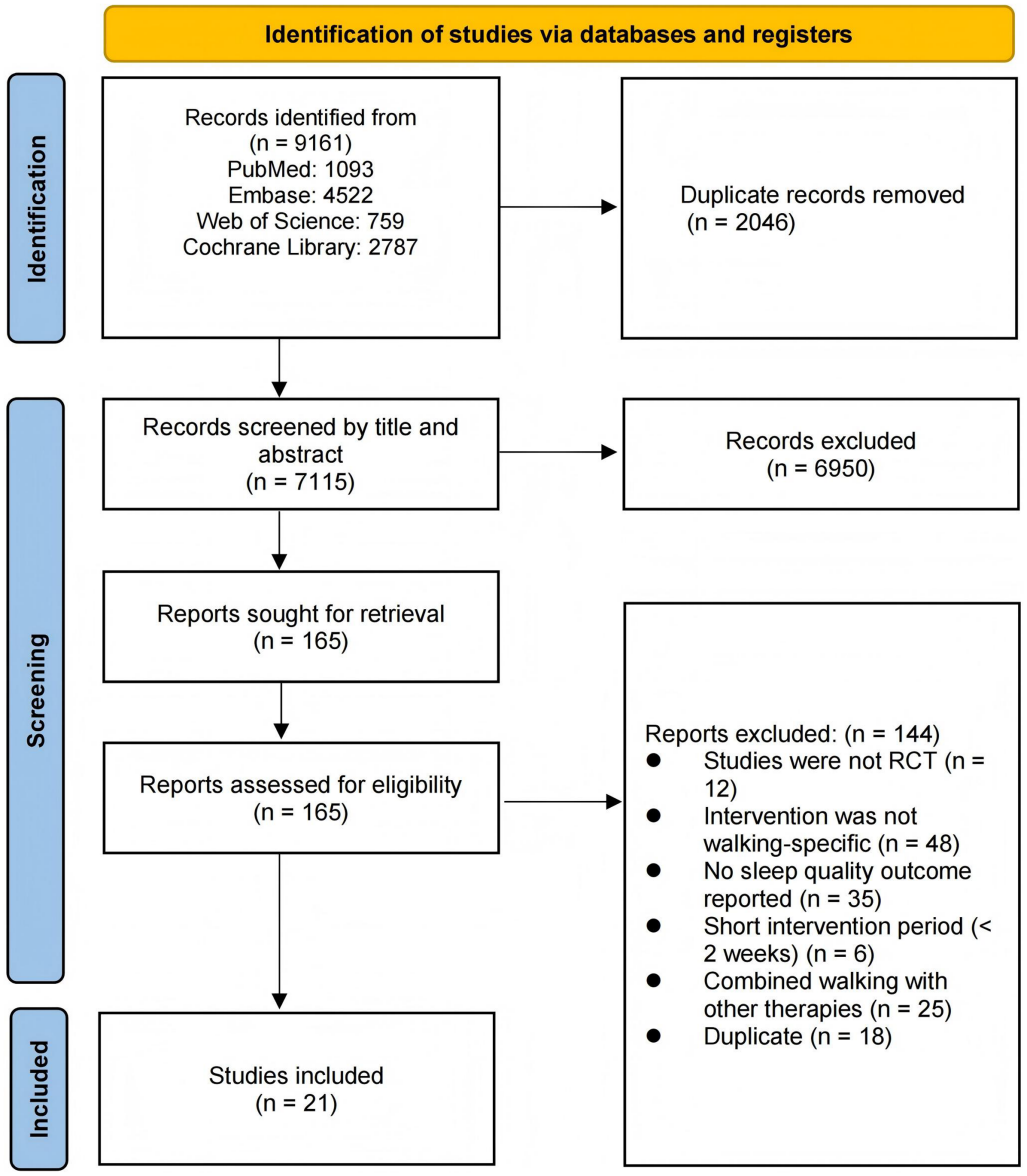
PRISMA flow diagram. A total of 9,161 records were identified from PubMed, Embase, the Cochrane Library, and Web of Science. After duplicate removal, 7,115 records were screened, and 21 randomized controlled trials were included in the Bayesian three-level meta-analysis.

A total of 9,161 records were identified from four databases (PubMed 1,093, Embase 4,522, Cochrane Library 2,787, Web of Science 759). After removal of 2,046 duplicates, 7,115 records were screened by title and abstract, and 6,950 were excluded. Full-text assessment of 165 candidate articles led to the exclusion of 144 for the following reasons: non-RCT design (*n =* 12), intervention not primarily based on walking (*n =* 48), no sleep quality outcome reported (*n =* 35), intervention duration less than 2 weeks (*n =* 6), walking combined with other therapies (*n =* 25), and duplicate publications (*n =* 18). Twenty-one RCTs were included in the quantitative synthesis (Figure 1).

The 21 studies were published between 2002 and 2026 and enrolled a total of 1,707 participants (888 in walking groups, 819 in control groups) from nine countries. China (7 studies) and Iran (4 studies) were the most represented, followed by the United States (3 studies) and Hungary (2 studies). The studies covered diverse populations. Patients with sleep disorders were the most common (8 studies), followed by healthy adults (3), cancer patients (2), and women’s health populations (2). Exercise intensity ranged from 3.2 to 5.7 METs (mean 4.1 METs), all within the moderate-intensity range. Session duration ranged from 30 to 150 min, weekly frequency from one to seven sessions, and total intervention duration from 4 to 52 weeks (median 12 weeks). Thirteen studies employed supervised training. Exercise settings included outdoor environments (8 studies), facility-based venues (8), and home-based settings (5). The PSQI was the most commonly used outcome measure (18 studies), followed by the ISI (2 studies) and the SWED-QUAL Sleep scale (1 study). Detailed characteristics of included studies are presented in Supplementary File 2.

### 3.2 Risk of bias

Seventeen studies were rated as having some concerns for overall risk of bias, and four were rated as high risk. The primary concerns were concentrated in two domains: outcome measurement (self-report instruments without blinding) and deviations from intended interventions (open-label designs). No study was rated as low risk. The influence of risk of bias on the pooled effect was further examined in sensitivity analysis (see Section 3.6; Supplementary File 15).

### 3.3 Pooled effect size

The Bayesian three-level random-effects model was fitted to 66 effect sizes from 21 studies. All MCMC diagnostic indicators confirmed adequate convergence (R^ range [1.000, 1.001], Bulk ESS ≥ 4,500; Supplementary File 3), and posterior predictive checks indicated satisfactory model fit (Supplementary File 6 and Supplementary Figure S1).

**Figure 2:**
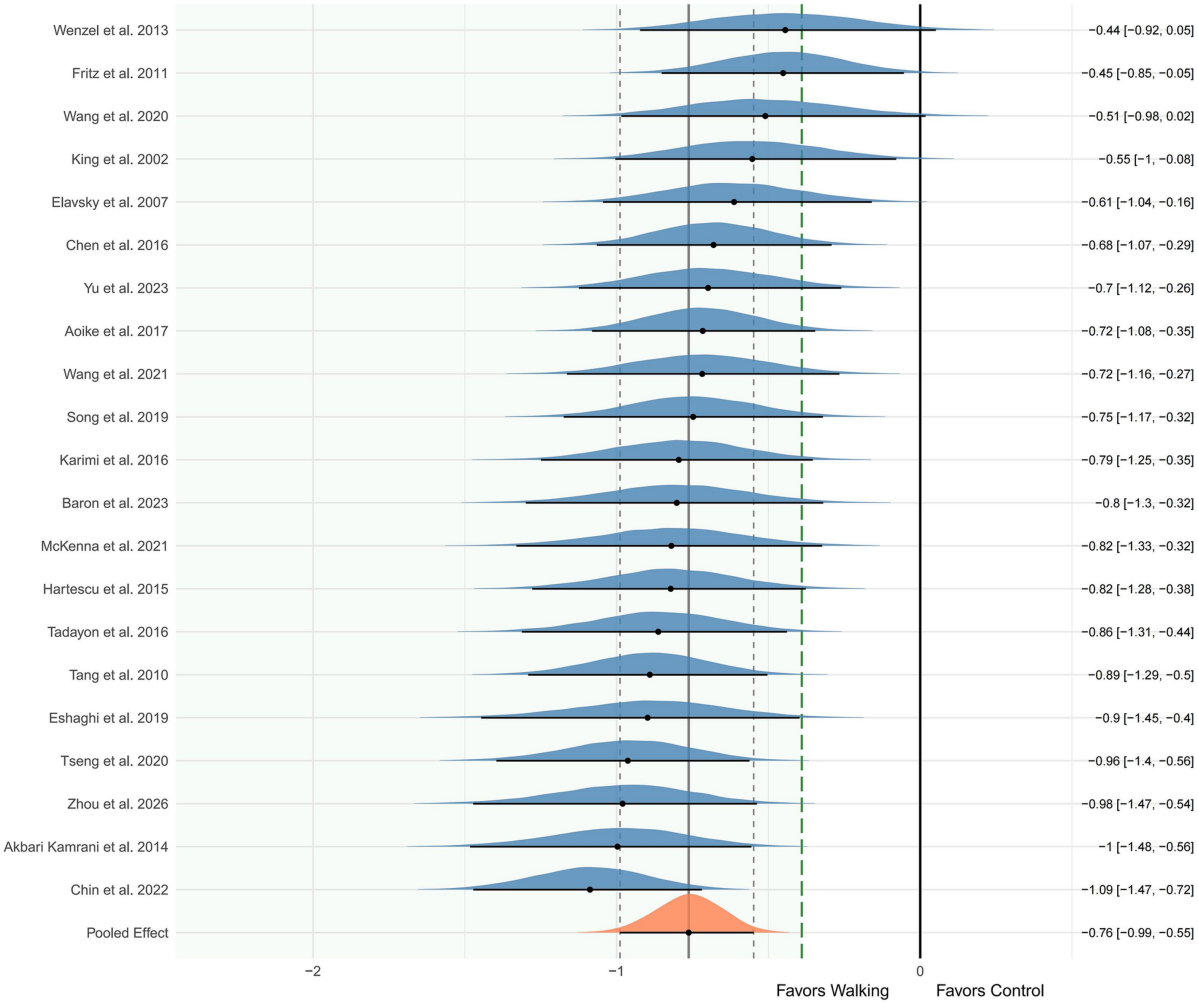
Forest plot of the effect of walking exercise on sleep quality (Hedges’ *g*). Each row represents the posterior effect size estimate and 95% credible interval (CrI) for an individual study. The orange ridge density and point estimate indicate the pooled effect (*g* = −0.76, 95% CrI [−0.99, −0.55]). The green dashed line with light green shading denotes the minimal clinically important difference (MCID = −0.39) [5]. The gray dashed line marks the null effect. Negative values favor the walking group over the control group.

**Figure 3:**
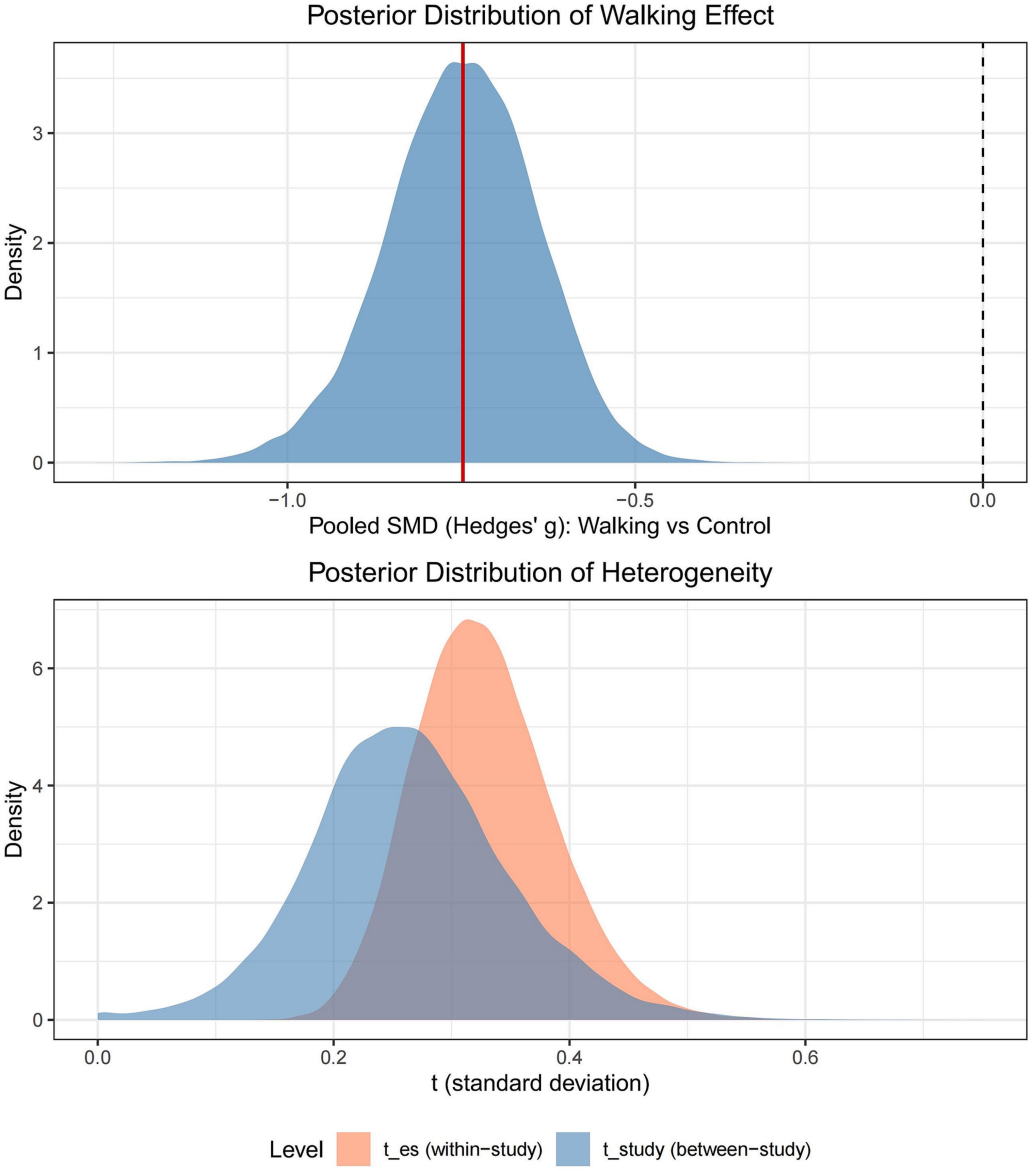
Posterior density plots. The left panel shows the posterior density of the pooled effect size (Hedges’ *g*) for walking, with a peak near −0.75 and the entire distribution falling below zero (pd = 100%). The right panel displays the posterior densities of the heterogeneity parameters: the blue line represents within-study heterogeneity *τ*_es_ (posterior median 0.33), and the red line represents between-study heterogeneity τ_study_ (posterior median 0.26).

Compared with control conditions, walking exercise produced a medium-to-large improvement in sleep quality (Hedges’ g = −0.76, 95% CrI [−0.99, −0.55]; Figure 2). The entire posterior distribution fell below zero (pd = 100%), with posterior probabilities of 100% for the effect exceeding −0.2, 99.2% for exceeding −0.5, and 31.5% for exceeding −0.8 (Figure 3; Supplementary File 4). The Savage–Dickey density ratio at the parameter level yielded BF_10_ = 8.16 × 10^5^, and bridge sampling at the model level yielded BF_10_ = 4.32 × 10^7^. Both values constituted extreme evidence in favor of the effect. ROPE analysis confirmed that 0% of the posterior fell within [−0.1, 0.1] or [−0.2, 0.2], and the 95% HDI was [−0.96, −0.54] (Supplementary File 5).

The estimated between-study heterogeneity standard deviation (τ_study) was 0.27 (95% CrI [0.11, 0.46]), and the within-study heterogeneity standard deviation (τ_es) was 0.34 (95% CrI [0.23, 0.47]). The total I^2^ was 73.7% (95% CrI [61.5, 83.8%]), indicating substantial heterogeneity. The within-study component (I^2^_es = 43.7%) exceeded the between-study component (I^2^_study = 28.7%), suggesting that the primary source of heterogeneity was variation among multiple effect sizes within the same study rather than variation across studies. The Bayesian R^2^ was 0.47 (95% CrI [0.35, 0.62]; Supplementary File 18). A summary of primary results is presented in Table 1, and posterior effect size estimates for individual studies are provided in Supplementary File 19. Grading of Recommendations Assessment, Development and Evaluation (GRADE) assessment rated the certainty of evidence as very low, with downgrading attributable to risk of bias, inconsistency, and publication bias (Table 1).

**Table 1:** Summary of Bayesian meta-analysis results for the effect of walking interventions on sleep quality.

Parameter	Value
*Pooled effect estimate*	
Number of studies (k)	21
Number of effect sizes	66
Hedges’ g [95% CrI]	−0.76 [−0.99, −0.55]
95% Prediction interval	[−1.37, −0.16]
Probability of direction (pd)	1.0000
P(effect < 0)	1.0000
P(effect ≤ MCID)	0.9997
*Heterogeneity*	
τ_study (between-study) [95% CrI]	0.27 [0.11, 0.46]
τ_es (within-study) [95% CrI]	0.34 [0.23, 0.47]
I^2^ total	73.7%
Bayesian R^2^ [95% CrI]	0.47 [0.35, 0.62]
*Bayes factors*	
BF₁₀ parameter (Savage-Dickey)	8.16 × 10^5^
BF₁₀ model (bridgesampling)	4.32 × 10^7^
*Clinical significance*	
MCID threshold (Hedges’ g)	−0.39
Source	Liang et al. (5)
P(Walking effect ≤ MCID)	99.97%
*Publication bias*	
Egger’s regression test (*p*)	< 0.001
Begg’s rank correlation test (*p*)	< 0.001
Fail-safe N (Rosenthal)	523
Fail-safe N threshold (5 k + 10)	200
*Sensitivity analyses*	
Prior sensitivity (Δg across 5 priors)	0.041
Prior influence on results	Negligible
*Evidence certainty (GRADE)*	
Risk of bias	Serious (−1)ᵃ
Inconsistency	Serious (−1)ᵇ
Indirectness	Not serious
Imprecision	Not serious
Publication bias	Serious (−1)ᶜ
Overall certainty	Very low

CrI, credible interval; PI, prediction interval; pd, probability of direction; MCID, minimum clinically important difference [(5), Sleep Medicine Reviews, 86, 102239]; BF₁₀, Bayes factor in favour of H₁; τ, standard deviation of random effects; I^2^, proportion of variability due to heterogeneity; R^2^, Bayesian coefficient of determination; GRADE, Grading of Recommendations Assessment, Development and Evaluation. Model: smd | se(se_smd) ~ 1 + group + (1 | studyID/es_id). Priors: Intercept ~ N(0, 1), b ~ N(0, 1), sd ~ Cauchy(0, 1). ᵃNo study rated as low risk of bias; 4 rated as high risk. ᵇI^2^ = 73.7%, indicating substantial heterogeneity, although 95% PI remained entirely below zero. ᶜEgger’s test *p* < 0.001; Begg’s test *p* < 0.001.

### 3.4 Clinical relevance and prediction interval

Using Hedges’ g ≤ −0.39 as the MCID threshold, the posterior probability of the pooled effect exceeding this threshold was 99.97%. At the individual study level, all 21 studies had posterior probabilities of exceeding the MCID above 58%, with 16 exceeding 90% (Supplementary File 7 and Supplementary Figure S2). The 95% prediction interval was [−1.37, −0.16], lying entirely below zero. These results indicate a 98.9% probability that a future comparable study would observe an effect favoring walking and an 89.6% probability of reaching the MCID threshold (Supplementary File 16 and Supplementary Figure S8).

### 3.5 Moderator analyses

**Figure 4:**
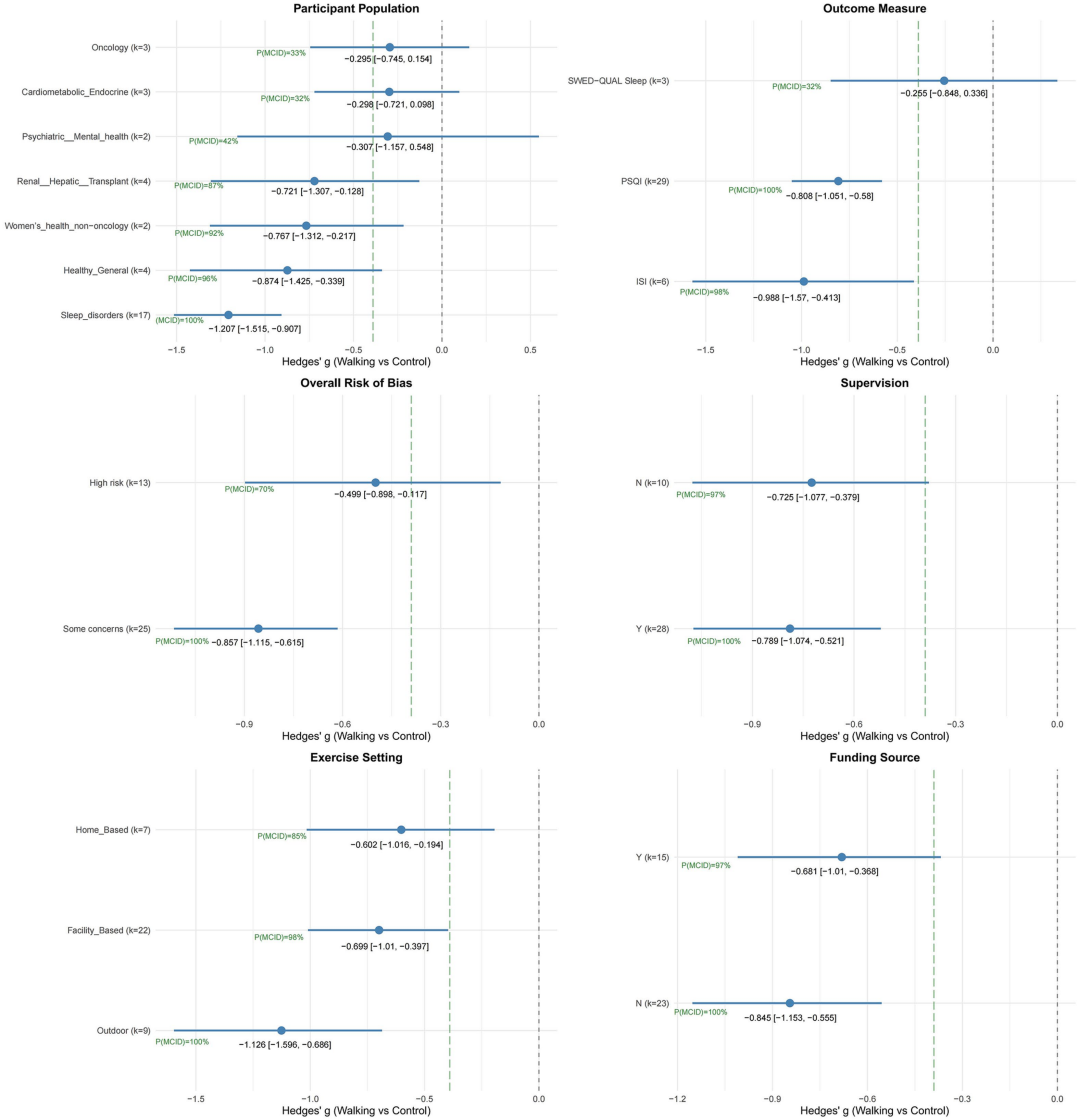
Subgroup analysis forest plot. Effect size estimates and 95% credible intervals are presented for six categorical moderators (population type, outcome measurement instrument, overall risk of bias, supervision mode, exercise setting, and funding source). The posterior probability of exceeding the MCID, P(MCID), is annotated beside each subgroup. Patients with sleep disorders showed the largest effect [*g* = −1.21, P(MCID) = 100%]. Population type was the only moderator with some evidence supporting between-subgroup differences (interaction BF = 4.27).

Subgroup analysis identified population type as the only moderator with some evidence supporting between-subgroup differences (interaction BF = 4.27). The largest effect was observed in patients with sleep disorders (g = −1.21, 95% CrI [−1.52, −0.91], P(MCID) = 100%), followed by healthy adults (g = −0.87) and renal/hepatic/transplant patients (g = −0.72). The interaction BFs for the remaining five moderators were all below 1 (outcome instrument 0.818, risk of bias 0.808, supervision mode 0.241, exercise setting 0.388, funding source 0.328), indicating that the data favored the null hypothesis of no between-subgroup differences. For the outcome instrument, although the interaction Bayes factor did not support a between-subgroup difference, the per-instrument estimates were heterogeneous: effects were strong for the ISI (g = −0.99, k = 6) and PSQI (g = −0.81, k = 29) but small and uncertain for the SWED-QUAL Sleep scale (g = −0.26, 95% CrI [−0.85, 0.34], k = 3), the credible interval of which crossed zero. Although outdoor walking showed the numerically largest effect (g = −1.13), the interaction BF did not support a meaningful difference across exercise settings (Figure 4; Supplementary File 8).

**Figure 5:**
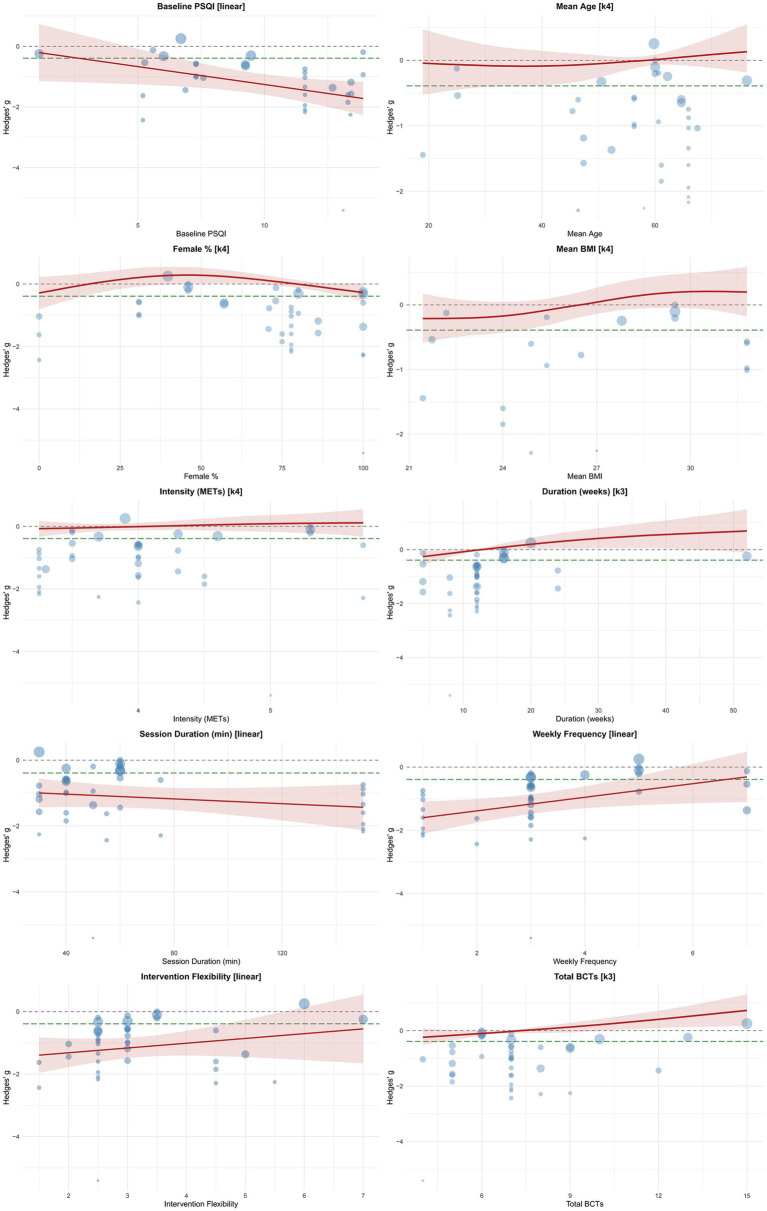
Moderator conditional effect plots. Each panel shows the walking effect size as a function of one of the 10 continuous moderators, fitted with the optimal model selected by LOO-IC. Brackets indicate the optimal model type (linear = linear, k3 = penalized spline with basis dimension k = 3, k4 = penalized spline with basis dimension k = 4). Shaded bands represent 95% credible intervals. The green dashed line marks the MCID (−0.39). Baseline PSQI score showed the strongest linear moderating effect (*β* = −0.302, BF₁₀ = 10.57), with worse baseline sleep quality associated with larger effects.

Among the continuous moderators examined through meta-regression, baseline PSQI score was the strongest predictor (linear model preferred, *β* = −0.302, 95% CrI [−0.486, −0.113], BF₁₀ = 10.57), with worse baseline sleep quality associated with larger effects. Weekly exercise frequency showed a linear association with effect size (β = 0.269, 95% CrI [0.096, 0.439], BF₁₀ = 6.76). The optimal models for intervention duration and total number of BCTs were both spline k = 3 (LOO-IC 86.94 and 88.87, respectively), suggesting nonlinear relationships with effect size. Mean age, proportion of female participants, BMI, and exercise intensity all favored spline k = 4 models, but their linear slope 95% CrIs included zero and BF₁₀ values were below 1, providing no evidence of a meaningful moderating effect. Session duration and intervention flexibility favored linear models but similarly lacked evidence of moderation (BF₁₀ < 1; Figure 5; Supplementary Files 9, 10 and Supplementary Figure S3). On the basis of these moderator findings, stratified walking prescription recommendations were developed (Table 2).

**Table 2:** Evidence-based walking prescription for sleep quality improvement derived from meta-analytic findings.

Parameter	Recommendation	Practical conversion	Evidence basis
*Target population*			
Primary indication	Adults with subjective sleep complaints (PSQI > 5)	Applicable to adults with subjective sleep complaints assessed by validated instruments (e.g., PSQI, ISI)	Pooled g = −0.76 [−0.99, −0.55]; BF₁₀ = 8.16 × 10^5^; pd. = 100%; P(MCID) = 99.97%
Priority population	Individuals with higher baseline PSQI scores (≥ 9, based on conventional PSQI scoring thresholds) benefit most	Each 1 SD increase in baseline PSQI (≈ 3.3 points) predicts an additional 0.30 SD reduction in post-intervention sleep disturbance	Meta-regression: β = −0.302, 95% CrI [−0.486, −0.113], BF₁₀ = 10.57 (strong evidence); Subgroup: sleep disorder population g = −1.21
*Intensity*			
Target METs	3.5 to 5.0 METs (moderate intensity)	Walking speed ≈ 4.8 to 6.4 km/h; heart rate ≈ 40 to 59% HRR (64 to 76% HRmax); RPE 12 to 13 (Borg 6–20 scale)	Observed range across 21 RCTs: 3.2 to 5.7 METs (mean 4.1); all within moderate-intensity domain per American College of Sports Medicine (ACSM) classification. METs assigned per 2024 Adult Compendium (18)
Perceived exertion cue	“Able to talk but not sing” (Talk Test)	Breathing noticeably increased; slight sweating; can maintain conversation	Talk Test corresponds to ventilatory threshold and moderate intensity (Reed & Pipe, 2014)
*Frequency and duration*			
Weekly frequency	3 to 5 sessions per week	E.g., Monday/Wednesday/Friday or every other day; accumulate ≥ 150 min moderate walking per week	Most included studies used 3 sessions/week (median); frequency showed a linear association (β = 0.269, BF₁₀ = 6.76), with higher frequencies associated with smaller effect estimates. Directionality warrants caution given the narrow observed range; 3–5 sessions/week is aligned with WHO guidelines and commonly used protocols.
Session duration	30 to 60 min per session	Include 3 to 5 min warm-up (slow walking) and 3 to 5 min cool-down; main bout 20 to 50 min at target intensity	Observed range: 30 to 75 min (excluding one outlier at 150 min); median 50 min across included studies
Programme length	Minimum 8 weeks; optimal 12–24 weeks	Initial benefits may appear by week 4 (3 studies); sustained improvement requires ≥ 8 weeks; cumulative meta-analysis showed effect stability from ~10 studies onward (mostly ≥ 12 weeks)	Duration showed non-linear relationship (best model: spline k = 3, LOO-IC = 86.94); 18 of 21 studies used ≥ 8 weeks
Estimated step count	3,000 to 7,000 steps per session; 9,000 to 35,000 steps per week	At 4.8 km/h (3.5 METs): ≈90–100 steps/min × 30 min ≈ 3,000 steps; at 6.4 km/h (5.0 METs): ≈120–130 steps/min × 60 min ≈ 7,000 steps. Weekly: 3–5 sessions × 3,000–7,000 steps	Cadence thresholds derived from Tudor-Locke et al. (39): 100 steps/min as moderate-intensity criterion. Step length estimated at 0.65–0.75 m based on speed and population norms
*Delivery mode*			
Supervision	Both supervised and unsupervised programmes are effective; choose based on feasibility	Supervised: group classes, instructor-led outdoor walks; Unsupervised: self-paced home walking with pedometer or phone app	Supervised g = −0.79 vs. unsupervised g = −0.73; interaction BF = 0.241 (evidence supports no difference)
Setting	Any accessible setting; outdoor walking showed numerically largest effect	Outdoor: parks, walking trails, campus paths; Facility: treadmill, indoor track; Home: neighbourhood walking	Outdoor g = −1.13, facility g = −0.70, home g = −0.60; interaction BF = 0.388 (no confirmed difference)
*Monitoring and progression*			
Progression strategy	Start at lower end of intensity and duration; increase by 10% per week	Week 1–2: 20 min at 3.5 METs (4.8 km/h); Week 3–4: 30 min at 4.0 METs; Week 5+: 40–60 min at 4.0–5.0 METs	Gradual progression consistent with ACSM guidelines for previously inactive adults

Recommendations are translated from the Bayesian three-level meta-analysis of 21 RCTs (*n =* 1,707). MCID = −0.39 (5). Practical conversions use the 2024 Adult Compendium of Physical Activities (18) and ACSM Guidelines for Exercise Testing and Prescription (11th ed.). METs, Metabolic Equivalents of Task; PSQI, Pittsburgh Sleep Quality Index; HRR, heart rate reserve; HRmax, maximum heart rate; RPE, rating of perceived exertion; BF₁₀, Bayes factor; CrI, credible interval; β, standardised regression coefficient; BCT, Behavior Change Technique. Step count estimates are based on cadence-intensity thresholds [(39), Br J Sports Med] and average step length norms; actual values vary with height, gait pattern, and terrain.

### 3.6 Sensitivity analyses

Leave-one-out analysis showed that removing any single study altered the pooled effect by −0.074 to +0.056. All estimates retained 95% CrIs excluding zero, confirming that the main finding held (Supplementary File 13 and Supplementary Figure S6). The pre-post correlation sensitivity analysis yielded pooled effects ranging from −0.739 to −0.763 across assumed r values of 0.3 to 0.9 (Δg = 0.024; Supplementary File 14 and Supplementary Figure S7). After excluding the four studies rated as high risk of bias, the pooled effect increased slightly in magnitude (g = −0.843, 95% CrI [−1.121, −0.579]), suggesting that high-risk studies attenuated rather than inflated the observed effect (Supplementary File 15). The prior sensitivity analysis demonstrated that pooled estimates varied by only 0.041 across five prior specifications ranging from Normal(0, 0.5) to Normal(0, 10), with P(MCID) ≥ 0.9995 under all specifications. These findings confirm that the results were driven by the data rather than by prior assumptions (Supplementary File 12 and Supplementary Figure S5).

### 3.7 Publication bias and evidence stability

The funnel plot displayed visible asymmetry (Supplementary File 11 and Supplementary Figure S4). Egger’s test (z = −6.53, *p* < 0.001) and Begg’s test (*τ* = −0.458, *p* < 0.001) both indicated publication bias or small-study effects. The fail-safe N was 523, far exceeding the 5 k + 10 = 200 threshold, indicating that the overall conclusion was resistant to unpublished null results. Cumulative meta-analysis showed that the pooled effect stabilized after approximately 10 studies had been included (around 2017). The final estimate based on all 21 studies (g = −0.765) was consistent with earlier accumulation stages. Both the direction and magnitude of the effect have stabilized with the current evidence base (Supplementary File 17 and Supplementary Figure S9).

## 4 Discussion

### 4.1 Main findings

The Bayesian three-level random-effects model showed that walking produced a medium-to-large improvement in subjective sleep quality among adults. Bayes factors provided extreme evidence in favor of this effect, and the posterior probability of reaching the MCID was 99.97%. The 95% prediction interval fell entirely below zero, indicating that even after accounting for between-study heterogeneity, a future comparable trial would likely observe a favorable effect. Heterogeneity decomposition revealed that most heterogeneity arose from differences among walking arms, outcome instruments, or time points within the same study, rather than from differences between studies.

Among moderators, baseline sleep quality emerged as the strongest predictor of effect magnitude, and weekly exercise frequency also showed a linear association with effect size. Bayes factors for supervision mode and exercise setting were well below 1, indicating that the current data favored the hypothesis of no meaningful differences in effectiveness across delivery formats. GRADE assessment rated the overall certainty of evidence as very low, with downgrading attributable to risk of bias, inconsistency, and publication bias. Four sets of sensitivity analyses and the cumulative meta-analysis confirmed the robustness of the conclusions with respect to both the direction of the effect and its clinical relevance.

### 4.2 Comparison with previous studies

Recent network meta-analyses and dose–response studies that examined walking as an independent exercise modality have reported effect sizes ranging from g = −0.63 to SMD = −0.807 (5, 8, 11, 12). The present estimate falls within this range and is slightly larger than estimates from analyses covering multiple exercise modalities (5). For insomnia-specific outcomes, walking or jogging ranked first among all exercise protocols in existing comparisons (11), and walking showed comparable or favorable efficacy relative to mind–body exercises such as tai chi and yoga across different outcome dimensions (40). The present review included only walking RCTs and excluded other exercise modalities, resulting in lower intervention heterogeneity, which may partly explain the slightly larger effect estimate compared with mixed-modality meta-analyses.

Methodologically, the present study is complementary to existing Bayesian dose–response studies (12–14). Those studies advanced nonlinear dose–response methodology in the exercise-sleep field but covered multiple exercise modalities and did not provide walking-specific dependent effect size handling or MCID exceedance probabilities. Our analysis extends these efforts by applying a three-level model to handle the hierarchical structure of 66 dependent effect sizes, decomposing heterogeneity into between-study and within-study components, and reporting clinical relevance in terms of posterior probabilities. With respect to the MCID, previous studies have predominantly adopted threshold-based evaluations, treating the question of whether an effect crosses a given threshold as a dichotomous judgment (6, 9, 12). The present study adopted an externally derived threshold and addressed clinical relevance in probabilistic terms, providing information more closely aligned with the uncertainty inherent in clinical decision-making.

The finding that baseline sleep quality was the strongest moderator is consistent with prior evidence. Large-scale analyses have identified baseline PSQI score as the most important effect moderator (5), and a similar pattern of larger effects among those with worse baseline sleep has been observed in older adults (12). The optimal models for intervention duration and total number of BCTs were both spline k = 3, consistent with nonlinear dose–response patterns reported elsewhere (5, 12, 14) and suggesting that exercise prescriptions for sleep improvement are unlikely to follow simple linear relationships. The specific shape of the nonlinearity, however, remains uncertain, with reports of U-shaped (5, 14), J-shaped (13), and other functional forms. The present findings support the presence of nonlinearity but cannot determine the precise functional form, indicating that further characterization will require additional well-designed RCTs.

The heterogeneity decomposition from the three-level model revealed that within-study variation exceeded between-study variation, a finding that has received limited attention in existing exercise-sleep meta-analyses. Effect size variability arose more from differences among walking arms, outcome measures, or assessment time points within individual studies than from systematic differences in populations, countries, or intervention protocols across studies. Previous meta-analyses have typically reported only total I^2^ without hierarchical decomposition (7–9) and therefore could not distinguish between these sources of heterogeneity. This finding has implications for future RCT design. Researchers should prioritize standardized reporting of walking prescription parameters, implementation details, and adherence, and should aim to unify outcome measurement instruments and assessment time windows (41) to reduce avoidable methodological heterogeneity. For evidence synthesis, future meta-analyses should consider three-level or multilevel models to handle such dependency structures rather than pooling or selecting a single effect size per study (23, 31).

### 4.3 Clinical implications

Clinical prioritization of walking interventions should be guided by the degree of baseline sleep impairment rather than disease diagnosis alone, as the Bayes factor for baseline PSQI score was larger than that for population type. The larger effects observed in sleep disorder populations likely reflect more severe baseline impairment and thus greater room for improvement. Mechanistically, walking may attenuate the circadian disruption, hyperarousal, and inflammation-related alterations common in insomnia (42) via several physiological pathways (43). Based on the current evidence, moderate-intensity walking performed three to five times per week, 30 to 60 min per session, for 12 to 24 weeks may provide a practical prescription window (Table 2). This corresponds to approximately 3,000 to 7,000 steps per session at cadences ranging from approximately 100 to 130 steps per minute across the moderate-intensity speed range (39), a metric supported by objective step-count evidence linking daily steps to sleep quality (44). The nonlinear dose–response findings suggest that the dose–response relationship may be nonlinear rather than monotonically increasing. The 95% prediction interval lying entirely below zero supports a high probability of benefit in future comparable studies. Supervision mode and exercise setting did not produce meaningfully different outcomes, suggesting that community-based and home-based programs may be compatible with broader dissemination. Whether outdoor walking confers additional circadian benefits through morning light exposure warrants further investigation (45).

### 4.4 Strengths and limitations

The main strengths of this study include its exclusive focus on walking as a single intervention modality, the use of a Bayesian three-level model to handle dependent effect sizes and decompose heterogeneity, data-driven comparison of linear and nonlinear moderator models, probabilistic reporting of clinical relevance, and multiple sensitivity analyses.

This study has several limitations. All included studies used self-report sleep measures and open-label designs, and none was rated as low risk of bias, so subjective reporting bias and expectancy effects cannot be excluded. Given that exercise tends to improve subjective sleep parameters more than objective ones (7), the effect sizes reported here may overestimate the true treatment effect. These concerns are reflected in the GRADE rating of very low certainty. The Bayesian indices and the GRADE rating address different questions. The Bayes factors and posterior probabilities quantify the strength of statistical evidence conditional on the included studies and the model assumptions, whereas GRADE appraises the certainty of the overall body of evidence by additionally accounting for risk of bias, inconsistency, and publication bias. This rating indicates uncertainty in the precise effect magnitude rather than absence of benefit, as the extreme Bayes factors and stable sensitivity analyses support the directional conclusion. Egger’s and Begg’s tests indicated publication bias or small-study effects. Although the fail-safe N far exceeded the threshold, this does not fully eliminate such influence. Some subgroups had small sample sizes, limiting generalizability. The included studies spanned nine countries, and differences in cultural context, climate, and healthcare systems may affect external validity.

## 5 Conclusion

The present evidence suggests that walking may improve subjective sleep quality in adults, with an effect of medium-to-large magnitude and a 99.97% posterior probability of reaching the minimal clinically important difference, although the overall certainty of evidence is very low and awaits confirmation from higher-quality RCTs. Individuals with worse baseline sleep quality derive larger effects. Favorable effects were consistent across supervision modes and settings, with no format emerging as clearly superior. Based on the current evidence, moderate-intensity walking performed three to five times per week, 30–60 min per session, for 12 to 24 weeks may serve as a practical non-pharmacological option, particularly for those with poorer baseline sleep. Higher-quality RCTs that incorporate objective sleep measures, test comparative or combined protocols, and assess real-world feasibility in community and primary care settings are needed.

## Data Availability

The original contributions presented in the study are included in the article/Supplementary material, further inquiries can be directed to the corresponding author.
