# Supplementary material for: Walking and subjective sleep quality in adults: a Bayesian three-level meta-analysis with probabilistic clinical relevance assessment and dose–response modeling
Source: Front Public Health. 2026 Jun 15;14:1864607. doi: 10.3389/fpubh.2026.1864607 (PMC13310748; doi:10.3389/fpubh.2026.1864607)
Supplement: Supplementary file 1 [file Data_Sheet_1.docx]

**Supplementary Materials**

**Table of Contents**

Supplementary File 1. Search Strategy

Supplementary File 2. Characteristics of Included Studies

Supplementary File 3. Model Specification and MCMC Diagnostics

Supplementary File 4. Posterior Distribution Summary Statistics

Supplementary File 5. Bayesian Inference Parameters

Supplementary File 6. Posterior Predictive Check

Supplementary File 7. MCID Probability Analysis

Supplementary File 8. Subgroup Analyses

Supplementary File 9. Meta-Regression Results (Linear vs Non-linear Model Comparison)

Supplementary File 10. Meta-Regression Coefficient Forest Plot

Supplementary File 11. Publication Bias Assessment

Supplementary File 12. Prior Sensitivity Analysis

Supplementary File 13. Leave-One-Out Sensitivity Analysis

Supplementary File 14. Pre-Post Correlation Sensitivity Analysis

Supplementary File 15. Risk of Bias Sensitivity Analysis

Supplementary File 16. Prediction Interval

Supplementary File 17. Cumulative Meta-Analysis

Supplementary File 18. Bayesian R²

Supplementary File 19. Study-Level Posterior Estimates

**Supplementary File 1. Search Strategy**

Search date: All databases searched from inception to March 2026.

**Database 1: PubMed**

#1 "Walking"[Mesh] OR "Walk*"[tiab] OR "ambulat*"[tiab] OR "brisk walk*"[tiab] OR "treadmill walk*"[tiab] OR "Nordic walk*"[tiab]

#2 "Sleep"[Mesh] OR "Sleep Wake Disorders"[Mesh] OR "Sleep Initiation and Maintenance Disorders"[Mesh] OR "sleep quality"[tiab] OR "sleep disturbance*"[tiab] OR "sleep disorder*"[tiab] OR "insomnia"[tiab] OR "Pittsburgh Sleep Quality Index"[tiab] OR "PSQI"[tiab] OR "sleep efficiency"[tiab] OR "sleep latency"[tiab] OR "sleep duration"[tiab] OR "Insomnia Severity Index"[tiab] OR "ISI"[tiab]

#3 "Randomized Controlled Trial"[pt] OR "Controlled Clinical Trial"[pt] OR "randomized"[tiab] OR "randomised"[tiab] OR "placebo"[tiab] OR "randomly"[tiab] OR "trial"[tiab] OR "RCT"[tiab]

#4 #1 AND #2 AND #3

**Database 2: Embase (via Embase.com)**

#1 'walking'/exp OR walk*:ti,ab OR ambulat*:ti,ab OR 'brisk walking':ti,ab OR 'treadmill walking':ti,ab OR 'nordic walking':ti,ab

#2 'sleep'/exp OR 'sleep disorder'/exp OR 'insomnia'/exp OR 'sleep quality':ti,ab OR 'sleep disturbance*':ti,ab OR 'sleep disorder*':ti,ab OR insomnia:ti,ab OR 'pittsburgh sleep quality index':ti,ab OR psqi:ti,ab OR 'sleep efficiency':ti,ab OR 'sleep latency':ti,ab OR 'sleep duration':ti,ab OR 'insomnia severity index':ti,ab OR isi:ti,ab

#3 'randomized controlled trial'/exp OR 'controlled clinical trial'/exp OR randomized:ti,ab OR randomised:ti,ab OR placebo:ti,ab OR randomly:ti,ab OR trial:ti,ab OR rct:ti,ab

#4 #1 AND #2 AND #3

**Database 3: Cochrane Library**

#1 MeSH descriptor: [Walking] explode all trees

#2 walk* OR ambulat* OR (brisk NEXT walk*) OR (treadmill NEXT walk*) OR (Nordic NEXT walk*):ti,ab,kw

#3 #1 OR #2

#4 MeSH descriptor: [Sleep] explode all trees

#5 MeSH descriptor: [Sleep Wake Disorders] explode all trees

#6 (sleep NEXT quality) OR (sleep NEXT disturbance*) OR (sleep NEXT disorder*) OR insomnia OR "Pittsburgh Sleep Quality Index" OR PSQI OR (sleep NEXT efficiency) OR (sleep NEXT latency) OR (sleep NEXT duration) OR "Insomnia Severity Index" OR ISI:ti,ab,kw

#7 #4 OR #5 OR #6

#8 #3 AND #7 (Limited to Trials)

**Database 4: Web of Science (Core Collection)**

TS=(walk* OR ambulat* OR "brisk walking" OR "treadmill walking" OR "Nordic walking") AND TS=("sleep quality" OR "sleep disturbance*" OR "sleep disorder*" OR "insomnia" OR "Pittsburgh Sleep Quality Index" OR "PSQI" OR "sleep efficiency" OR "sleep latency" OR "sleep duration" OR "Insomnia Severity Index" OR "ISI") AND TS=("randomized" OR "randomised" OR "randomly" OR "controlled trial" OR "RCT" OR "placebo" OR "clinical trial")

**Supplementary File 2. Characteristics of Included Studies**

The full characteristics of all 21 included studies are presented in this section, including author, year, country, population type, sample size (Walking/Control), mean age, sex distribution, baseline PSQI score, exercise prescription (intensity in METs, session duration, weekly frequency, and total duration in weeks), exercise setting, supervision status, outcome measure, and overall risk of bias (RoB 2) rating. Exercise intensity was expressed as Metabolic Equivalents of Task (METs) and, when not directly reported in the original study, was assigned using the 2024 Adult Compendium of Physical Activities (Herrmann et al., 2024).

| **Author** | **Year** | **Country** | **Population** | **N**  **(W/C)** | **Age**  **(years)** | **Female**  **(%)** | **Baseline PSQI** | **Exercise Prescription**  **(METs × min × freq × duration)** | **Setting** | **Supervised** | **Outcome** | **Overall RoB** |
| --- | --- | --- | --- | --- | --- | --- | --- | --- | --- | --- | --- | --- |
| Abby CKing | 2002 | USA | Other Occupational | 45/45 | 62.2 | 100% | 1.1 | 4.3 METs × 40 min × 4/wk × 52 wks | Home Based | Yes | PSQI | Some concerns |
| Ahmad Ali Akbari Kamrani | 2014 | Iran | Healthy General | 30/15 | NR | 0% | 5.2 | 4 METs × 55 min × 2/wk × 8 wks | Outdoor | Yes | PSQI | Some concerns |
| Dan Song | 2019 | China | Neurologic | 60/60 | 76.2 | 80% | 9.5 | 4.6 METs × 60 min × 3/wk × 16 wks | Facility Based | Yes | PSQI | Some concerns |
| Danilo Takashi Aoike | 2017 | Brazil | Renal Hepatic Transplant | 50/30 | 56.3 | 31% | 7.3 | 4 METs × 40 min × 3/wk × 12 wks | Facility Based | Yes | PSQI | Some concerns |
| Danny JYu | 2023 | China | Psychiatric Mental health | 20/10 | 60.6 | 80% | 13.9 | 3.5 METs × 50 min × 3/wk × 12 wks | Facility Based | Yes | PSQI | Some concerns |
| Edwin CChin | 2022 | China | Sleep disorders | 74/18 | 65.9 | 78% | 11.6 | 3.2 METs × 150 min × 1/wk × 12 wks | Facility Based | Yes | PSQI | High risk |
| Feifei Wang | 2020 | Hungary | Healthy General | 14/12 | 25 | 73% | 5.6 | 3.5 METs × 60 min × 7/wk × 4 wks | Outdoor | No | PSQI | High risk |
| Feifei Wang | 2021 | Hungary | Healthy General | 22/24 | 25.1 | 73% | 5.3 | 3.5 METs × 60 min × 7/wk × 4 wks | Outdoor | No | PSQI | High risk |
| Hui Mei Chen | 2016 | China | Oncology | 90/94 | 64.6 | 57% | 9.2 | 4 METs × 40 min × 3/wk × 12 wks | Home Based | No | PSQI | Some concerns |
| Iuliana Hartescu | 2015 | UK | Sleep disorders | 17/19 | 45.4 | 71% | NR | 4.3 METs × 30 min × 5/wk × 24 wks | Home Based | No | ISI | Some concerns |
| Jennifer AWenzel | 2013 | USA | Oncology | 68/58 | 59.8 | 40% | 6.7 | 3.9 METs × 30 min × 5/wk × 20 wks | Home Based | No | PSQI | Some concerns |
| MTadayon | 2016 | Iran | Women’s health non-oncology | 56/56 | 52.3 | 100% | 12.7 | 3.3 METs × 50 min × 7/wk × 12 wks | Facility Based | No | PSQI | Some concerns |
| Mei Feng Tang | 2010 | China | Sleep disorders | 72/70 | 47.4 | 86% | 13.4 | 4 METs × 30 min × 3/wk × 4 wks | Home Based | No | PSQI | Some concerns |
| Pauline Baron | 2023 | France | Sleep disorders | 24/24 | 46.4 | 100% | NR | 5.7 METs × 75 min × 3/wk × 12 wks | Outdoor | Yes | ISI | Some concerns |
| Saba Karimi | 2016 | Iran | Sleep disorders | 23/23 | 67.5 | 0% | 7.6 | 3.5 METs × 30 min × 3/wk × 8 wks | Facility Based | Yes | PSQI | Some concerns |
| Sean GMc Kenna | 2021 | Ireland | Pain Musculoskeletal Rheumatologic | 8/10 | 58 | 100% | 13.4 | 3.7 METs × 30 min × 4/wk × 8 wks | Outdoor | No | PSQI | Some concerns |
| Shahrian Eshaghi | 2019 | Iran | Sleep disorders | 9/9 | NR | 100% | 13.1 | 5 METs × 50 min × 3/wk × 8 wks | Outdoor | Yes | PSQI | Some concerns |
| Steriani Elavsky | 2007 | USA | Women’s health non-oncology | 55/55 | 50.5 | 100% | 6 | 3.7 METs × 60 min × 3/wk × 16 wks | Outdoor | Yes | PSQI | Some concerns |
| Tomas Fritz | 2011 | Sweden | Cardiometabolic Endocrine | 87/125 | 60 | 46% | NR | 5.3 METs × 60 min × 5/wk × 16 wks | Facility Based | Yes | SWED-QUAL Sleep | High risk |
| Tseng Hau Tseng | 2020 | China | Sleep disorders | 40/40 | 61.1 | 75% | 13.3 | 4.5 METs × 40 min × 3/wk × 12 wks | Facility Based | Yes | PSQI | Some concerns |
| Xiaoyun Zhou | 2026 | China | Sleep disorders | 24/22 | 19 | 71% | 6.9 | 4.3 METs × 60 min × 3/wk × 24 wks | Outdoor | Yes | PSQI | Some concerns |

*N (W/C) = sample size (Walking/Control); PSQI = Pittsburgh Sleep Quality Index; METs = Metabolic Equivalents of Task; RoB = Risk of Bias (RoB 2); NR = not reported. Exercise prescription formatted as: intensity × session duration × weekly frequency × total duration.*

**Supplementary File 3. Model Specification and MCMC Diagnostics**

**Model specification**

Formula: smd | se(se_smd) ~ 1 + group + (1 | studyID/es_id)

Family: Gaussian (identity link)

Priors: Intercept ~ Normal(0, 1); b ~ Normal(0, 1); sd ~ Cauchy(0, 1)

MCMC: 8 chains × 6,000 iterations (3,000 warmup); adapt_delta = 0.99

Total post-warmup draws: 24,000

Data: 66 effect sizes from 21 studies

**Fixed effects**

| **Parameter** | **Estimate** | **SE** | **95% CrI** | **Rhat** | **Bulk ESS** | **Tail ESS** |
| --- | --- | --- | --- | --- | --- | --- |
| Intercept | −0.08 | 0.10 | [−0.28, 0.12] | 1.00 | 13,937 | 16,521 |
| groupWalking | −0.76 | 0.11 | [−0.99, −0.55] | 1.00 | 13,140 | 16,146 |

**Random effects (hyperparameters)**

| **Level** | **Estimate** | **SE** | **95% CrI** | **Rhat** | **Bulk ESS** | **Tail ESS** |
| --- | --- | --- | --- | --- | --- | --- |
| τ_study (studyID) | 0.27 | 0.09 | [0.11, 0.46] | 1.00 | 5,456 | 5,539 |
| τ_es (studyID:es_id) | 0.34 | 0.06 | [0.23, 0.47] | 1.00 | 6,729 | 12,153 |

**MCMC convergence diagnostics**

All Rhat values < 1.01: TRUE

Rhat range: [1.000, 1.001]

Neff ratio range: [0.13, 0.85]

No divergent transitions were observed.

*MCMC, Markov chain Monte Carlo; ESS, effective sample size; Rhat, potential scale reduction factor; CrI, credible interval; SE, standard error; τ, standard deviation of random effects.*

**Supplementary File 4. Posterior Distribution Summary Statistics**

**Walking effect (b_groupWalking)**

| **Statistic** | **Value** |
| --- | --- |
| Mean | −0.762 |
| Median | −0.760 |
| SD | 0.112 |
| MAD | 0.111 |
| 95% CrI | [−0.989, −0.548] |
| 50% CrI | [−0.836, −0.687] |
| P(SMD < 0) | 1.0000 |
| P(SMD < −0.2) | 1.0000 |
| P(SMD < −0.5) | 0.9923 |
| P(SMD < −0.8) | 0.3573 |

**Heterogeneity (I²)**

| **Component** | **Median** | **95% CrI** |
| --- | --- | --- |
| I²_total | 73.7% | [61.5%, 83.8%] |
| I²_study (between-study) | 28.7% | [4.7%, 56.2%] |
| I²_es (within-study) | 43.7% | [22.3%, 69.2%] |

*CrI, credible interval; SMD, standardised mean difference; SD, standard deviation; MAD, median absolute deviation; I², proportion of variability due to heterogeneity; τ, heterogeneity parameter.*

**Supplementary File 5. Bayesian Inference Parameters**

**Bayes factors**

| **Test** | **BF₁₀** | **Interpretation** |
| --- | --- | --- |
| Parameter-level (Savage-Dickey): groupWalking | 8.16 × 10⁵ | Extreme evidence for H₁ |
| Model-level (bridgesampling): H₁ vs H₀ | 4.32 × 10⁷ | Extreme evidence for H₁ |

*Note: BF₁₀ > 100 indicates extreme evidence in favour of H₁ (Jeffreys, 1961).*

**ROPE analysis (Region of Practical Equivalence)**

The percentage of the posterior distribution falling inside the ROPE was computed for two ranges. For groupWalking, 0% of the posterior fell within ROPE [−0.1, 0.1] and 0% within ROPE [−0.2, 0.2], confirming that the walking effect is of practical significance and not negligible. For the Intercept, 57.74% fell within [−0.1, 0.1] and 91.10% within [−0.2, 0.2], consistent with the control group baseline being close to zero.

**Highest Density Interval (HDI)**

89% HDI for groupWalking: [−0.92, −0.58]; 95% HDI for groupWalking: [−0.96, −0.54]. Both intervals fall entirely outside the ROPE, providing strong evidence that the walking effect is practically meaningful. For the Intercept, the 89% HDI was [−0.24, 0.07] and the 95% HDI was [−0.27, 0.11].

*BF₁₀, Bayes factor in favour of H₁; pd, probability of direction; ROPE, Region of Practical Equivalence; HDI, Highest Density Interval; CrI, credible interval.*

**Supplementary File 6. Posterior Predictive Check**

The posterior predictive check compares the observed data distribution (dark line) with simulated data from the posterior predictive distribution (light lines). Good overlap indicates adequate model fit.


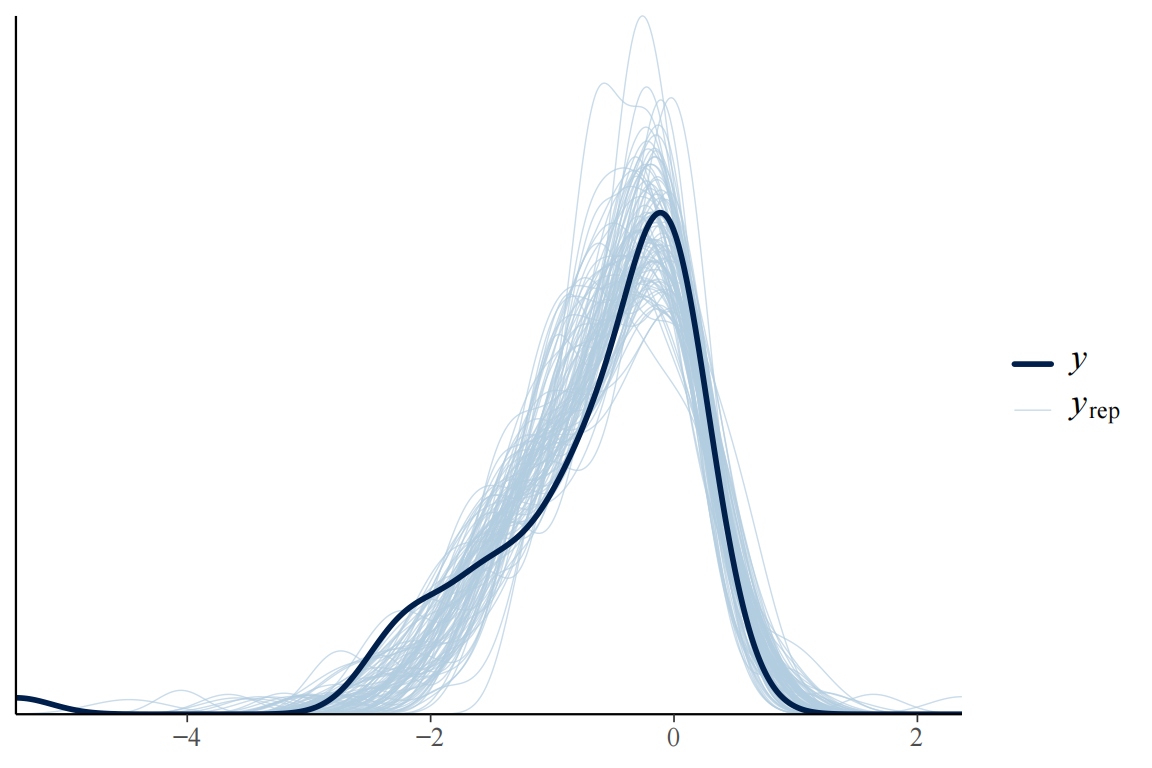


**Supplementary Figure S1.** Posterior predictive check of the main Bayesian model. The dark line represents the observed data distribution; light lines represent simulated datasets from the posterior predictive distribution.

**Supplementary File 7. MCID Probability Analysis**

The minimum clinically important difference (MCID) was set at Hedges' g = −0.39, sourced from Liang et al. (2026, Sleep Medicine Reviews, 86, 102239), based on a distribution-based method across 200 RCTs (n = 23,523) encompassing 14 exercise types.

Pooled P(Walking effect ≤ MCID) = 0.9996 (99.96%)

**Study-level MCID probabilities**

| **Study** | **P(effect ≤ MCID)** |
| --- | --- |
| EdwinCChin2022 | 1.0000 |
| AhmadAliAkbariKamrani2014 | 0.9972 |
| XiaoyunZhou2026 | 0.9954 |
| TsengHauTseng2020 | 0.9969 |
| MeiFengTang2010 | 0.9943 |
| ShahrianEshaghi2019 | 0.9755 |
| MTadayon2016 | 0.9843 |
| IulianaHartescu2015 | 0.9714 |
| SeanGMcKenna2021 | 0.9543 |
| SabaKarimi2016 | 0.9650 |
| PaulineBaron2023 | 0.9582 |
| DanSong2019 | 0.9494 |
| DaniloTakashiAoike2017 | 0.9570 |
| FeifeiWang2021 | 0.9275 |
| DannyJYu2023 | 0.9159 |
| HuiMeiChen2016 | 0.9321 |
| SterianiElavsky2007 | 0.8333 |
| AbbyCKing2002 | 0.7502 |
| FeifeiWang2020 | 0.6845 |
| TomasFritz2011 | 0.6073 |
| JenniferAWenzel2013 | 0.5826 |
| Pooled Effect | 0.9997 |


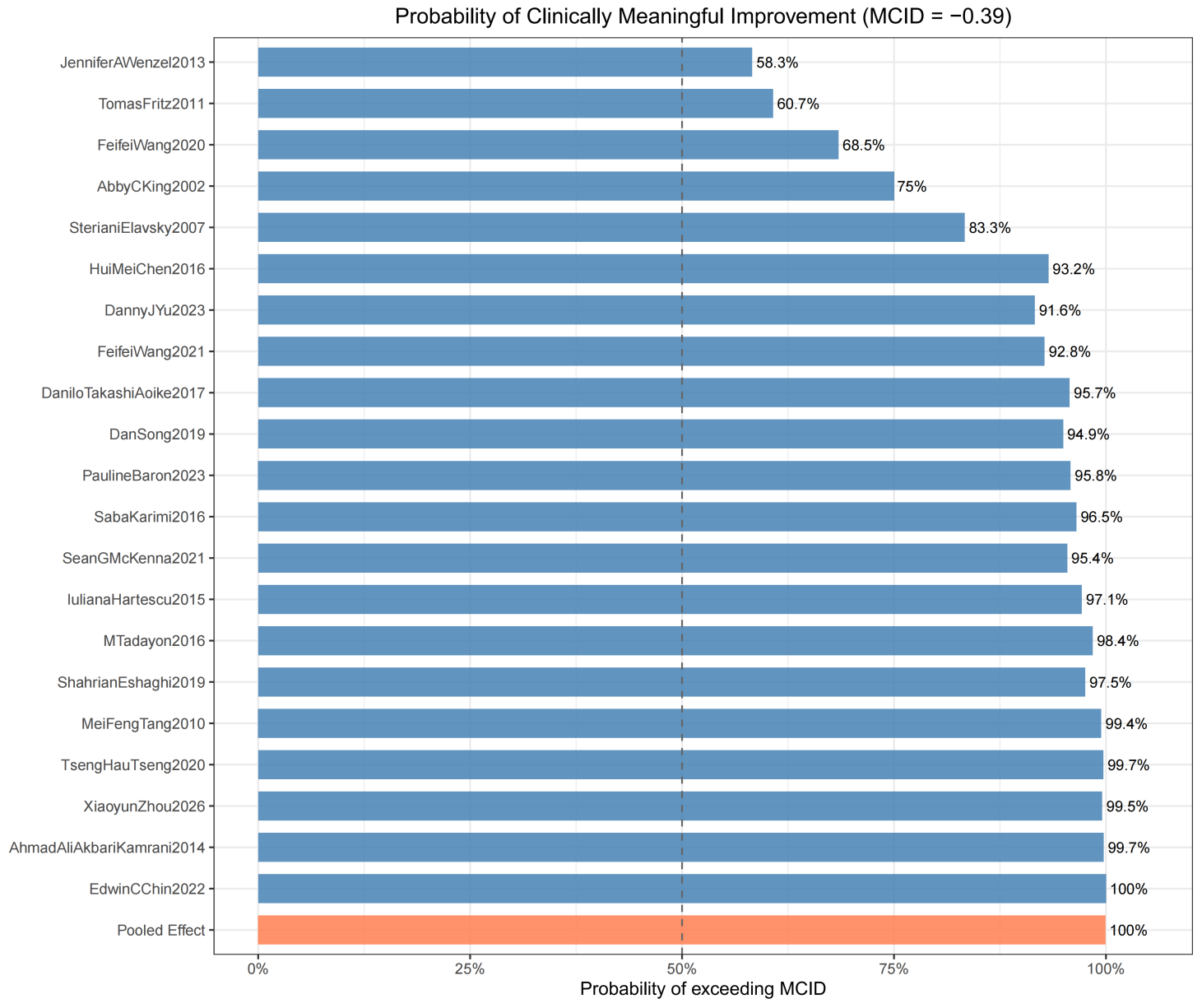


**Supplementary Figure S2.** Probability of each study's Walking effect exceeding the MCID threshold (Hedges' g ≤ −0.39).

**Supplementary File 8. Subgroup Analyses**

Subgroup analyses were conducted using interaction models (group × moderator). Bayes factors (BF) for the interaction term indicate evidence for or against subgroup differences. BF > 1 supports differences; BF < 1 supports no differences.

**Population type**

| **Subgroup** | **k** | **g [95% CrI]** | **pd** | **P(MCID)** | **Interaction BF** |
| --- | --- | --- | --- | --- | --- |
| Sleep disorders | 17 | −1.21 [−1.52, −0.91] | 1.0000 | 1.0000 |  |
| Healthy/General | 4 | −0.87 [−1.43, −0.34] | 0.9992 | 0.9621 |  |
| Renal/Hepatic/Transplant | 4 | −0.72 [−1.31, −0.13] | 0.9908 | 0.8682 |  |
| Psychiatric/Mental health | 2 | −0.31 [−1.16, 0.55] | 0.7610 | 0.4220 |  |
| Cardiometabolic/Endocrine | 3 | −0.30 [−0.72, 0.10] | 0.9234 | 0.3242 |  |
| Oncology | 3 | −0.30 [−0.75, 0.15] | 0.9086 | 0.3285 |  |
| Women's health | 2 | −0.77 [−1.31, −0.22] | 0.9961 | 0.9212 | 4.27 |

**Outcome measure**

| **Subgroup** | **k** | **g [95% CrI]** | **pd** | **P(MCID)** | **Interaction BF** |
| --- | --- | --- | --- | --- | --- |
| PSQI | 29 | −0.81 [−1.05, −0.58] | 1.0000 | 0.9996 |  |
| ISI | 6 | −0.99 [−1.57, −0.41] | 0.9995 | 0.9795 |  |
| SWED-QUAL Sleep | 3 | −0.26 [−0.85, 0.34] | 0.8039 | 0.3241 | 0.818 |

**Risk of bias**

| **Subgroup** | **k** | **g [95% CrI]** | **pd** | **P(MCID)** | **Interaction BF** |
| --- | --- | --- | --- | --- | --- |
| Some concerns | 25 | −0.86 [−1.12, −0.62] | 1.0000 | 1.0000 |  |
| High risk | 13 | −0.50 [−0.90, −0.12] | 0.9959 | 0.6970 | 0.808 |

**Supervision, Setting, Funding**

| **Moderator** | **Subgroup** | **k** | **g [95% CrI]** | **pd** | **P(MCID)** | **BF** |
| --- | --- | --- | --- | --- | --- | --- |
| Supervision | Yes | 28 | −0.79 [−1.07, −0.52] | 1.0000 | 0.9984 |  |
|  | No | 10 | −0.73 [−1.08, −0.38] | 1.0000 | 0.9706 | 0.241 |
| Setting | Outdoor | 9 | −1.13 [−1.60, −0.69] | 1.0000 | 0.9995 |  |
|  | Facility-based | 22 | −0.70 [−1.01, −0.40] | 1.0000 | 0.9781 |  |
|  | Home-based | 7 | −0.60 [−1.02, −0.19] | 0.9978 | 0.8534 | 0.388 |
| Funding | No | 23 | −0.85 [−1.15, −0.56] | 1.0000 | 0.9990 |  |
|  | Yes | 15 | −0.68 [−1.01, −0.37] | 1.0000 | 0.9656 | 0.328 |

*See Figure 5 in the main text for the subgroup forest plot.*

*k, number of effect sizes; g, Hedges' g; CrI, credible interval; pd, probability of direction; P(MCID), probability of exceeding the minimum clinically important difference; BF, Bayes factor for the interaction term; PSQI, Pittsburgh Sleep Quality Index; ISI, Insomnia Severity Index.*

**Supplementary File 9. Meta-Regression Results (Linear vs Non-linear Model Comparison)**

For each continuous moderator, three competing models were fitted: linear, a penalized spline with basis dimension k = 3, and a penalized spline with basis dimension k = 4. Here k denotes the dimension of the spline basis (the number of basis functions), with larger k permitting greater flexibility, rather than the number of knots. Model selection was based on LOO-IC (lower = better). Variables with fewer than 6 unique values were fitted with linear models only.

**Model comparison summary**

| **Moderator** | **n unique** | **β (linear)** | **95% CrI** | **pd** | **Best model** | **BF₁₀** |
| --- | --- | --- | --- | --- | --- | --- |
| Baseline PSQI | 18 | −0.302 | [−0.486, −0.113] | 0.999 | linear | — |
| Mean Age | 19 | 0.056 | [−0.136, 0.240] | 0.728 | k4 | — |
| Female % | 14 | −0.054 | [−0.254, 0.147] | 0.700 | k4 | — |
| Mean BMI | 11 | 0.179 | [−0.106, 0.455] | 0.901 | k4 | — |
| Intensity (METs) | 12 | 0.067 | [−0.166, 0.278] | 0.734 | k4 | — |
| Duration (weeks) | 7 | 0.182 | [0.020, 0.345] | 0.986 | linear | — |
| Session duration (min) | 7 | −0.148 | [−0.384, 0.091] | 0.892 | linear | — |
| Weekly frequency | 6 | 0.282 | [0.102, 0.463] | 0.998 | k3 | — |
| Intervention flexibility | 10 | 0.148 | [−0.027, 0.319] | 0.952 | k4 | — |
| Total BCTs | 10 | 0.209 | [0.050, 0.375] | 0.991 | linear | — |

*β = standardised slope (change in walking effect per 1 SD increase in the moderator). BF₁₀, Bayes factor for the linear interaction term (Savage-Dickey). LOO-IC, leave-one-out information criterion. pd, probability of direction. CrI, credible interval. METs, Metabolic Equivalents of Task. BMI, body mass index. BCTs, Behaviour Change Techniques. SMD, standardised mean difference. BF₁₀ = Bayes factor for the linear interaction term (Savage-Dickey). Best model selected by LOO-IC.*

**LOO-IC values**

| **Moderator** | **Linear** | **k = 3** | **k = 4** | **Best** |
| --- | --- | --- | --- | --- |
| Baseline PSQI | 73.7 | 74.3 | 75.5 | linear |
| Mean Age | 69.6 | 70.0 | 68.8 | k4 |
| Female % | 90.5 | 83.0 | 81.1 | k4 |
| Mean BMI | 46.0 | 47.4 | 45.7 | k4 |
| Intensity (METs) | 93.6 | 93.3 | 90.8 | k4 |
| Duration (weeks) | 86.8 | 87.7 | 89.9 | linear |
| Session duration (min) | 88.7 | 91.3 | 92.5 | linear |
| Weekly frequency | 85.4 | 85.1 | 86.1 | k3 |
| Intervention flexibility | 90.8 | 91.3 | 88.3 | k4 |
| Total BCTs | 87.1 | 87.3 | 90.4 | linear |

*See Figure 4 in the main text for the conditional effects plot.*

**Supplementary File 10. Meta-Regression Coefficient Forest Plot**


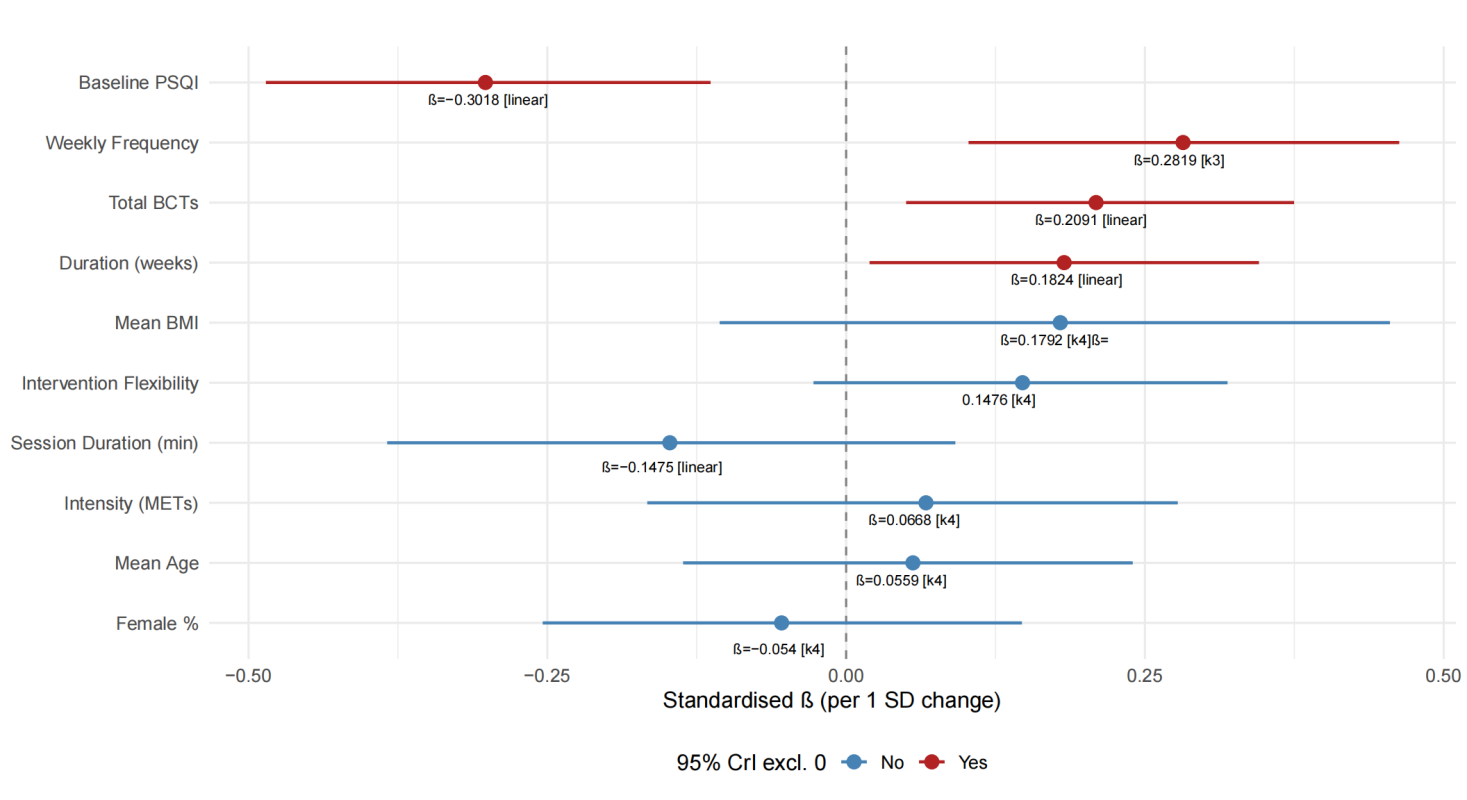


**Supplementary Figure S3.** Forest plot of standardised meta-regression coefficients (β) for all continuous moderators. Red indicates 95% CrI excluding zero.

**Supplementary File 11. Publication Bias Assessment**

| **Test** | **Statistic** | **p-value** | **Interpretation** |
| --- | --- | --- | --- |
| Egger's regression | z = −6.52 | < 0.001 | Significant asymmetry |
| Begg's rank correlation | τ = −0.454 | < 0.001 | Significant rank correlation |
| Fail-safe N (Rosenthal) | N = 523 | — | Exceeds 5k+10 = 200; robust |

Both Egger's and Begg's tests indicated significant funnel plot asymmetry, suggesting potential publication bias. However, the Fail-safe N of 523 substantially exceeded the threshold of 200 (5k + 10), indicating that the overall conclusion is robust to unpublished null results.

*k, number of effect sizes in the analysis (Walking arms = 39); 5k + 10, Rosenthal's threshold for robustness.*


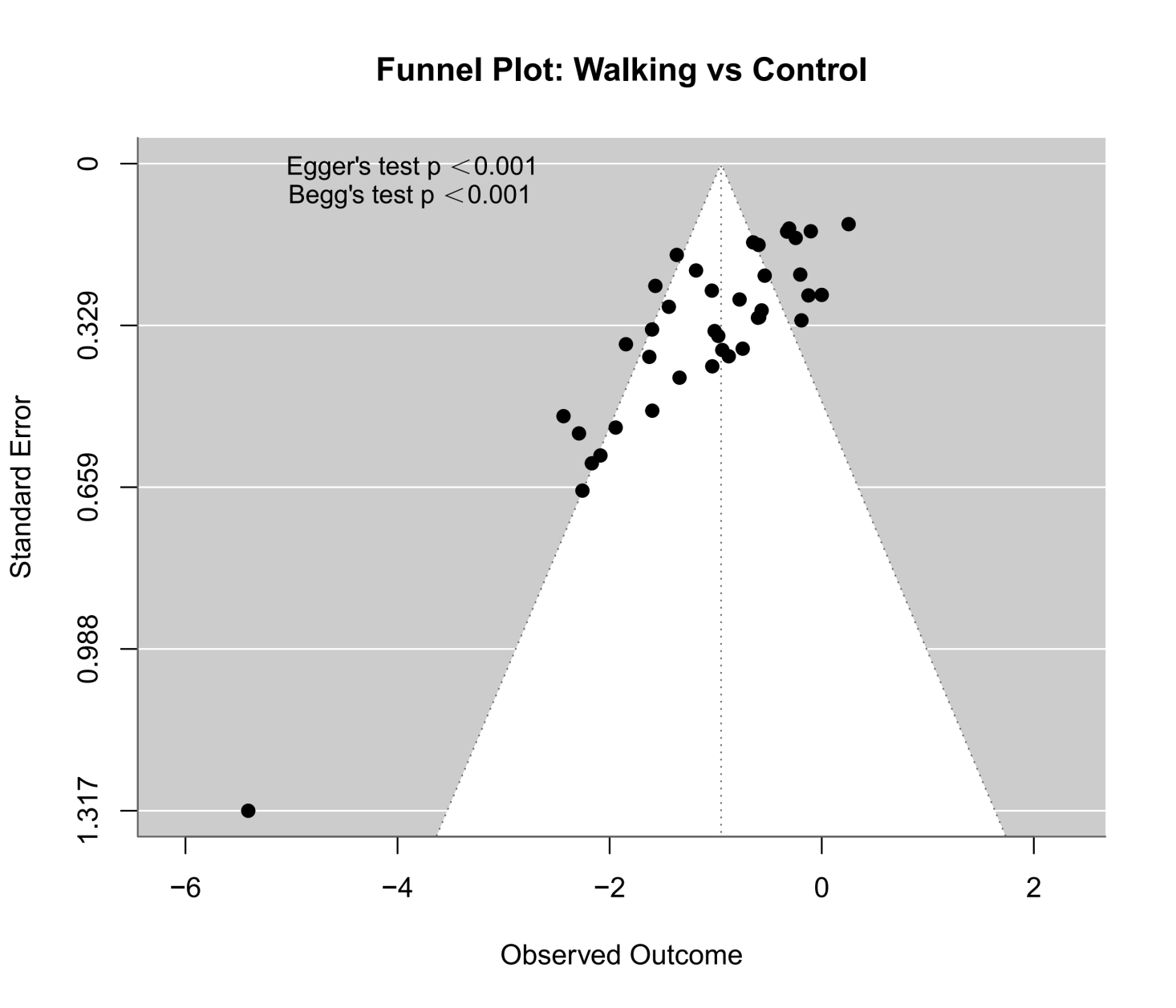


**Supplementary Figure S4.** Funnel plot of Walking arm effect sizes with Egger's and Begg's test results.

**Supplementary File 12. Prior Sensitivity Analysis**

Five sets of priors with varying widths were used to assess whether the posterior was driven by the prior or the data.

| **Prior** | **Estimate** | **95% CrI** | **pd** | **P(MCID)** |
| --- | --- | --- | --- | --- |
| Narrow: N(0, 0.5), C(0, 0.5) | −0.732 | [−0.951, −0.533] | 1.0000 | 0.9995 |
| Main: N(0, 1), C(0, 1) | −0.763 | [−0.989, −0.548] | 1.0000 | 0.9997 |
| Wide: N(0, 2), C(0, 2) | −0.773 | [−1.007, −0.559] | 1.0000 | 0.9996 |
| Very wide: N(0, 5), C(0, 5) | −0.773 | [−1.006, −0.562] | 1.0000 | 0.9996 |
| Extremely wide: N(0, 10), C(0, 10) | −0.772 | [−1.009, −0.554] | 1.0000 | 0.9996 |

Δg across all priors = 0.041, indicating high robustness. Results were data-driven rather than prior-driven.

*CrI, credible interval; pd, probability of direction; P(MCID), probability of exceeding the MCID; N, Normal distribution; C, Cauchy distribution; Δg, range of Hedges' g estimates across prior specifications.*


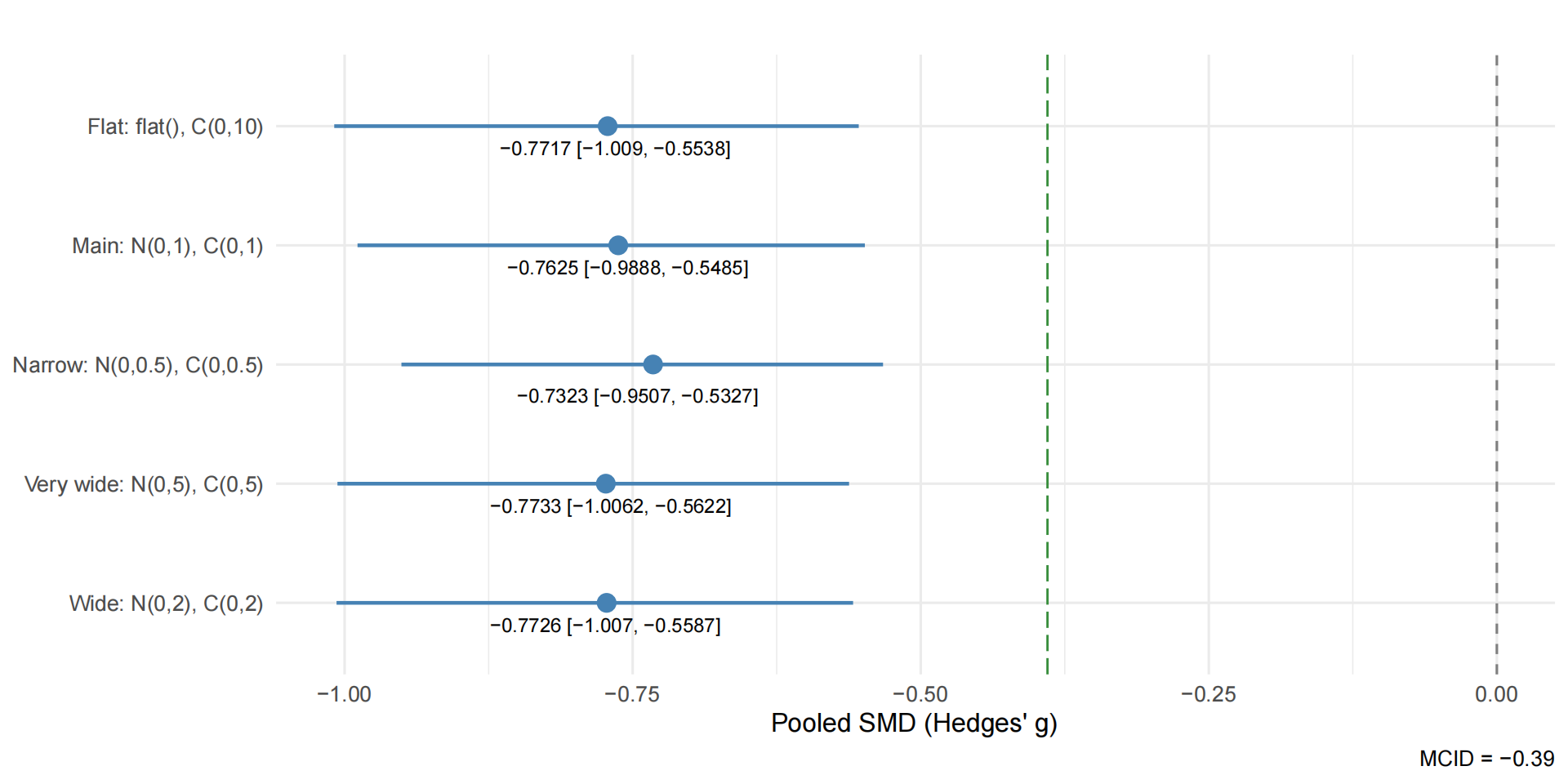


**Supplementary Figure S5.** Prior sensitivity analysis showing pooled Walking effect estimates under five prior specifications of varying widths.

**Supplementary File 13. Leave-One-Out Sensitivity Analysis**

Full model estimate: g = −0.763 [−0.989, −0.548]

| **Excluded Study** | **Estimate** | **95% CrI** |
| --- | --- | --- |
| None (Full Model) | −0.763 | [−0.989, −0.548] |
| SterianiElavsky2007 | −0.785 | [−0.999, −0.563] |
| ShahrianEshaghi2019 | −0.744 | [−0.943, −0.529] |
| FeifeiWang2020 | −0.774 | [−0.982, −0.555] |
| FeifeiWang2021 | −0.783 | [−0.998, −0.553] |
| SabaKarimi2016 | −0.755 | [−0.976, −0.536] |
| TsengHauTseng2020 | −0.708 | [−0.920, −0.488] |
| SeanGMcKenna2021 | −0.736 | [−0.941, −0.519] |
| MeiFengTang2010 | −0.709 | [−0.936, −0.490] |
| IulianaHartescu2015 | −0.779 | [−0.997, −0.556] |
| DanSong2019 | −0.799 | [−0.997, −0.582] |
| PaulineBaron2023 | −0.715 | [−0.934, −0.512] |
| JenniferAWenzel2013 | −0.795 | [−0.993, −0.582] |
| DannyJYu2023 | −0.783 | [−0.992, −0.560] |
| MTadayon2016 | −0.730 | [−0.953, −0.509] |
| HuiMeiChen2016 | −0.791 | [−1.013, −0.555] |
| AbbyCKing2002 | −0.784 | [−0.989, −0.557] |
| TomasFritz2011 | −0.843 | [−1.071, −0.595] |
| EdwinCChin2022 | −0.738 | [−0.961, −0.510] |
| AhmadAliAkbariKamrani2014 | −0.706 | [−0.909, −0.500] |
| DaniloTakashiAoike2017 | −0.776 | [−0.992, −0.537] |
| XiaoyunZhou2026 | −0.758 | [−0.972, −0.537] |

Maximum positive deviation: +0.056 (excluding AhmadAliAkbariKamrani2014). Maximum negative deviation: −0.074 (excluding TomasFritz2011). No single study substantially altered the pooled estimate.

*CrI, credible interval.*


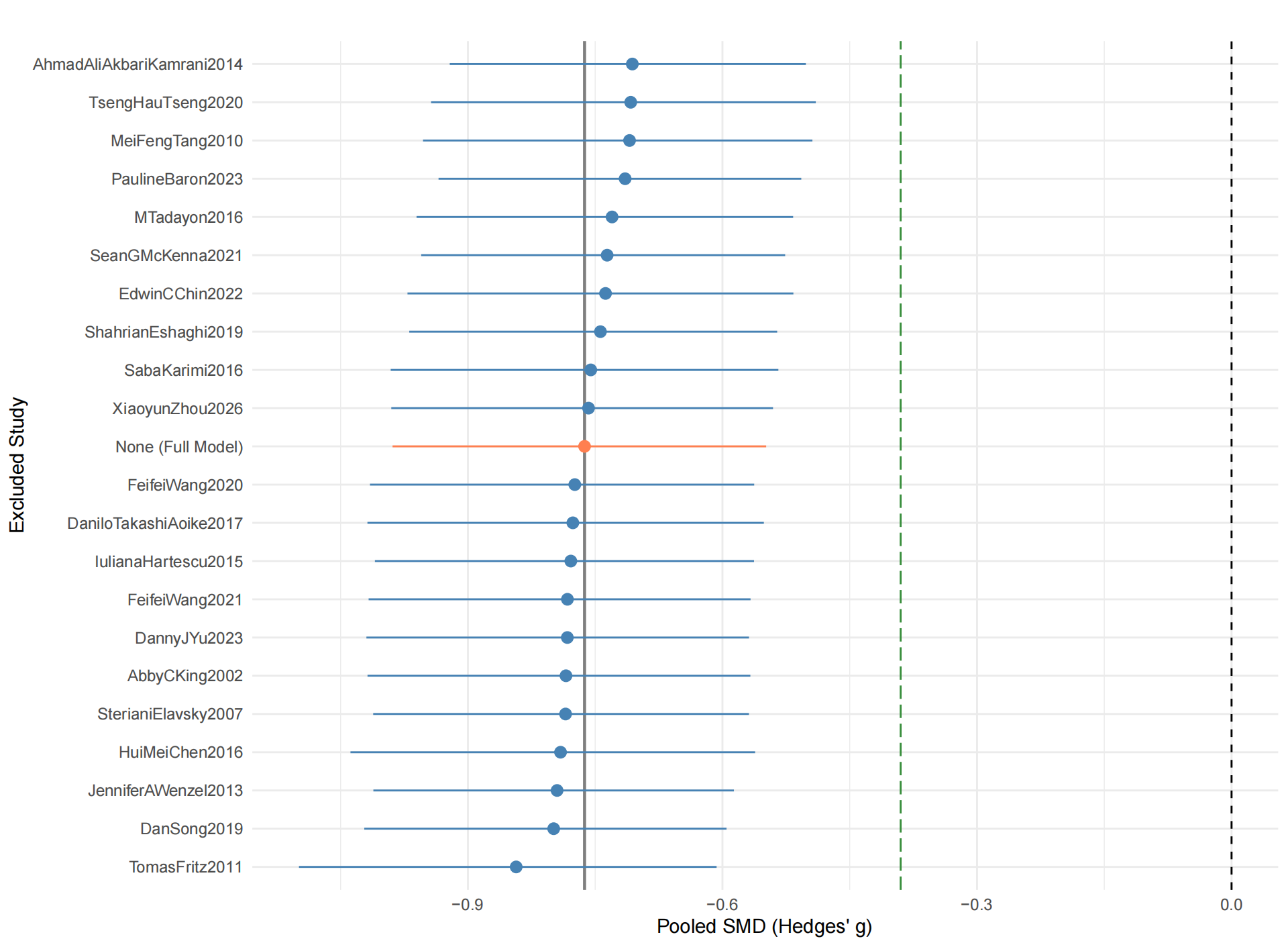


**Supplementary Figure S6.** Leave-one-out sensitivity analysis. Each point shows the pooled estimate after excluding one study. The coral point represents the full model.

**Supplementary File 14. Pre-Post Correlation Sensitivity Analysis**

The main analysis assumed r = 0.5 for the pre-post correlation when computing the standard error of Hedges' g.

| **r** | **Estimate** | **SE** | **95% CrI** |
| --- | --- | --- | --- |
| 0.3 | −0.738 | 0.109 | [−0.958, −0.534] |
| 0.5 (main) | −0.748 | 0.107 | [−0.968, −0.549] |
| 0.7 | −0.755 | 0.107 | [−0.973, −0.558] |
| 0.9 | −0.766 | 0.104 | [−0.972, −0.563] |

Δg = 0.024 across all correlation assumptions, indicating high robustness.


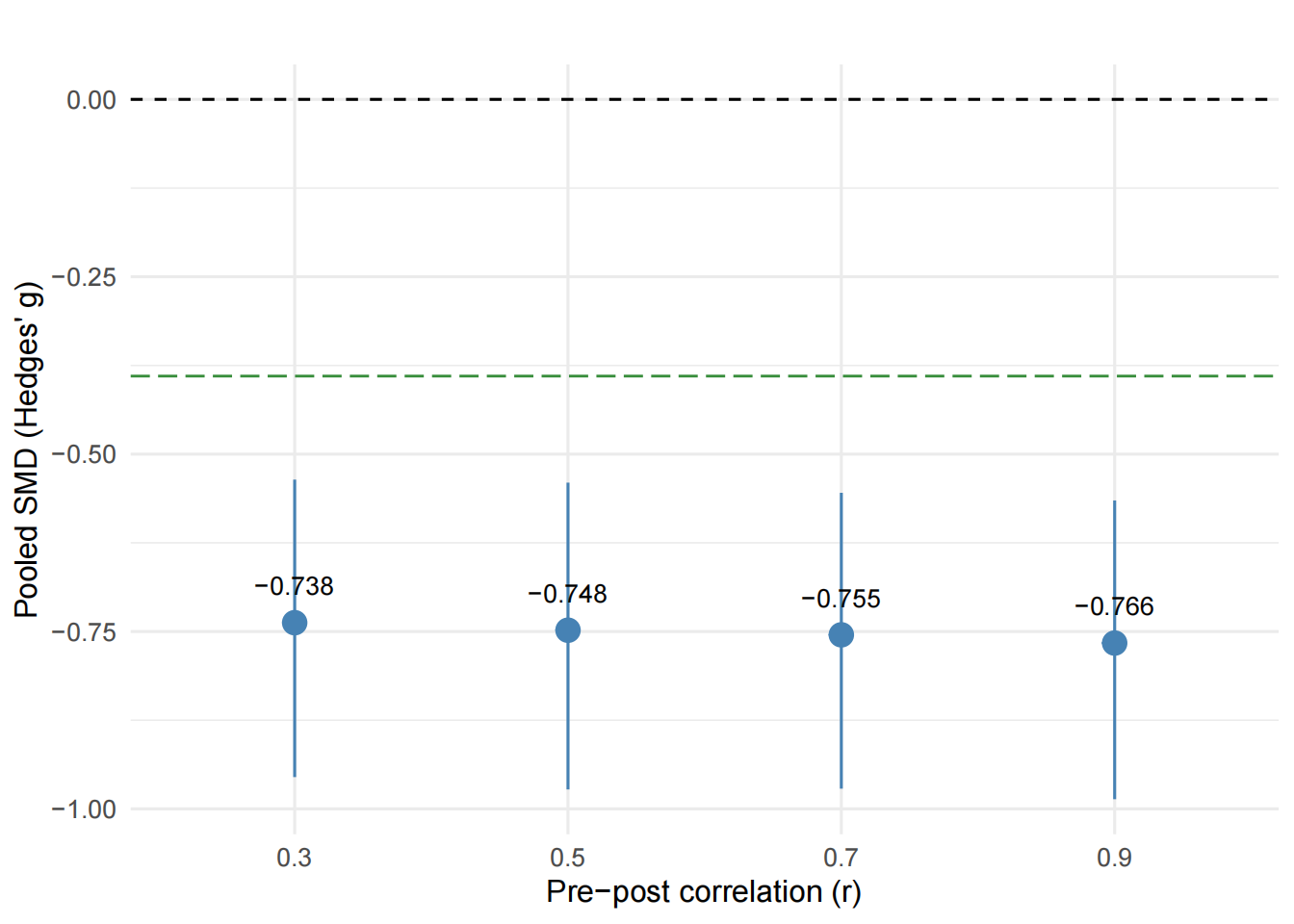


**Supplementary Figure S7.** Sensitivity analysis for assumed pre-post correlation (r = 0.3, 0.5, 0.7, 0.9).

**Supplementary File 15. Risk of Bias Sensitivity Analysis**

| **Subset** | **k** | **Estimate** | **95% CrI** |
| --- | --- | --- | --- |
| All studies | 21 | −0.763 | [−0.989, −0.548] |
| Excluding high risk | 17 | −0.862 | [−1.121, −0.579] |

Excluding studies rated as high risk of bias did not attenuate the pooled effect; the estimate was slightly larger in magnitude, suggesting that high-risk studies may have underestimated the effect.

*k, number of studies; CrI, credible interval.*

**Supplementary File 16. Prediction Interval**

The prediction interval estimates the expected range of effect sizes in a future new study, incorporating between-study heterogeneity (τ).

| **Metric** | **Value** |
| --- | --- |
| 95% Credible Interval (CrI) | [−0.989, −0.548] |
| 95% Prediction Interval (PI) | [−1.366, −0.156] |
| 50% Prediction Interval | [−0.951, −0.571] |
| P(new study effect < 0) | 0.989 |
| P(new study effect ≤ MCID) | 0.899 |

The prediction interval was wider than the credible interval, reflecting substantial between-study heterogeneity. Nevertheless, 89.6% of predicted new study effects would exceed the MCID threshold.

*CrI, credible interval; PI, prediction interval; MCID, minimum clinically important difference; τ, between-study heterogeneity parameter.*


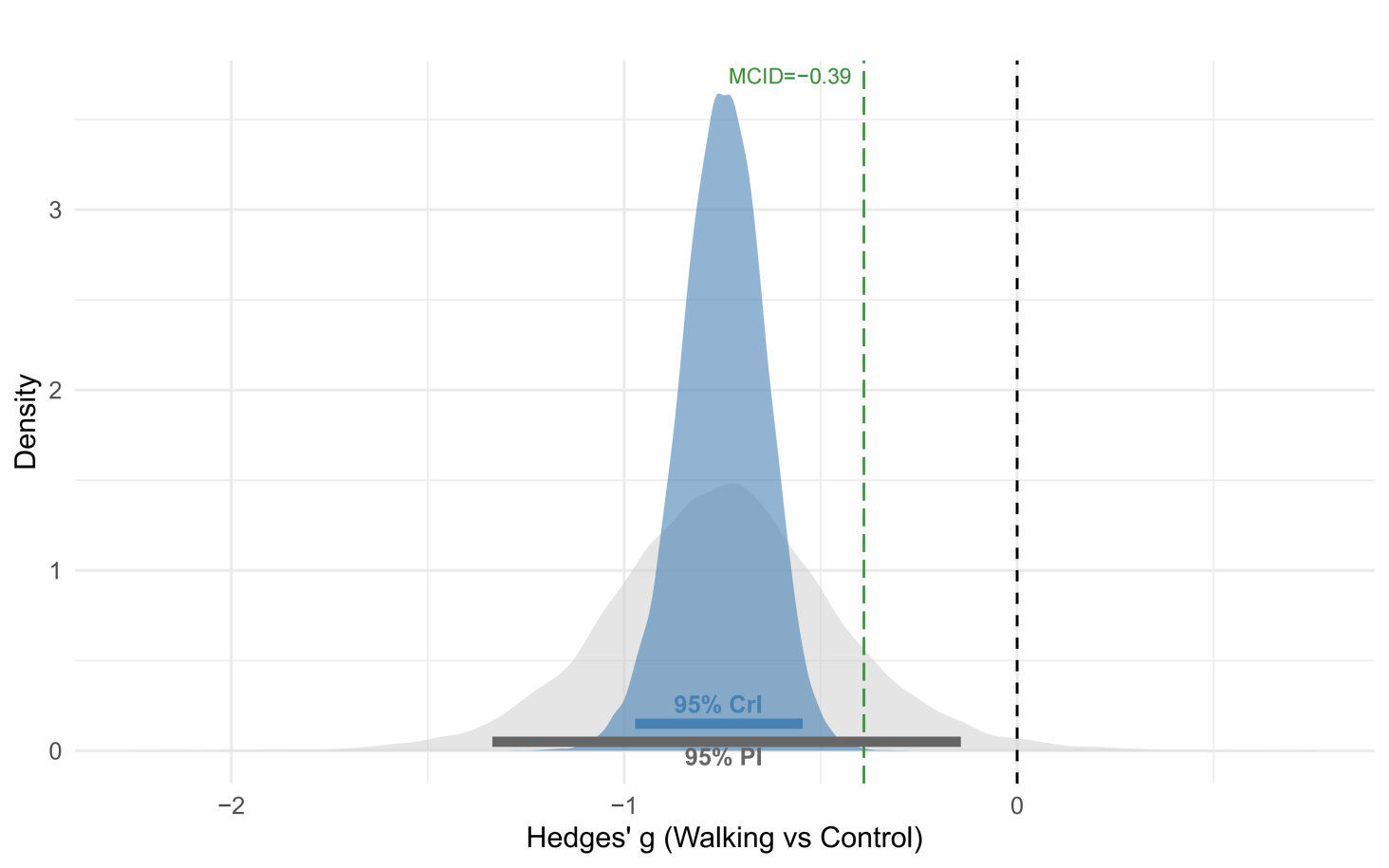


**Supplementary Figure S8.** Posterior distribution of the pooled Walking effect (blue) versus the prediction interval distribution (grey), with 95% CrI and 95% PI indicated.

**Supplementary File 17. Cumulative Meta-Analysis**

Studies were added sequentially by publication year. The pooled estimate stabilised after approximately 10 studies (circa 2017).

*k, cumulative number of studies; CrI, credible interval.*

| **k** | **Latest Study Added** | **Year** | **Estimate** | **95% CrI** |
| --- | --- | --- | --- | --- |
| 2 | SterianiElavsky2007 | 2007 | −0.319 | [−0.895, 0.407] |
| 3 | MeiFengTang2010 | 2010 | −0.728 | [−1.338, −0.040] |
| 5 | AhmadAliAkbariKamrani2014 | 2014 | −0.598 | [−1.050, −0.170] |
| 10 | DaniloTakashiAoike2017 | 2017 | −0.651 | [−0.918, −0.415] |
| 15 | TsengHauTseng2020 | 2020 | −0.693 | [−0.948, −0.446] |
| 18 | EdwinCChin2022 | 2022 | −0.731 | [−0.963, −0.515] |
| 20 | DannyJYu2023 | 2023 | −0.760 | [−0.958, −0.534] |
| 21 | XiaoyunZhou2026 | 2026 | −0.765 | [−0.969, −0.539] |


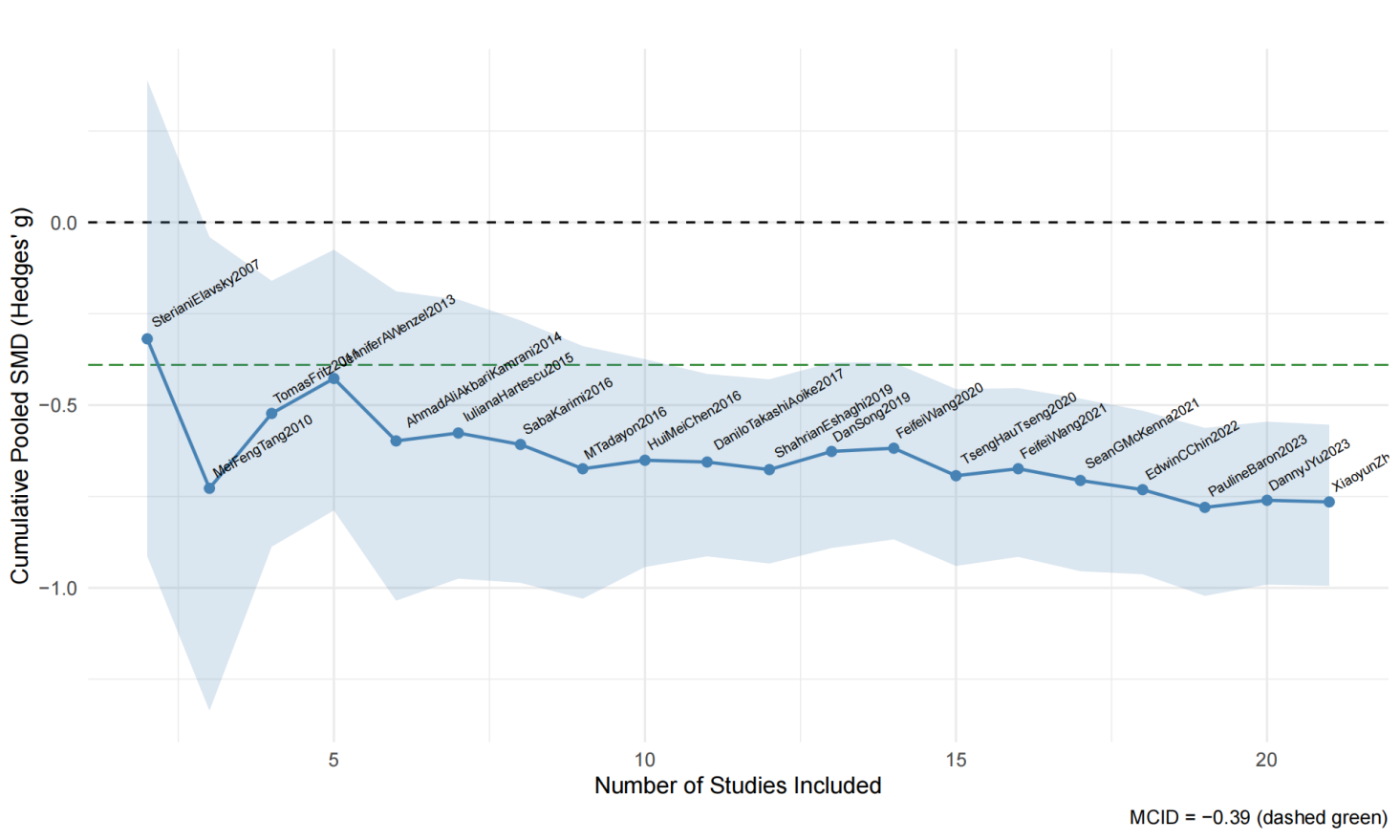


**Supplementary Figure S9.** Cumulative meta-analysis showing the evolution of the pooled estimate as studies were added chronologically.

**Supplementary File 18. Bayesian R²**

| **Metric** | **Estimate** | **SE** | **95% CrI** |
| --- | --- | --- | --- |
| R² | 0.475 | 0.070 | [0.345, 0.620] |

The model explained approximately 46.6% of the variance in observed effect sizes.

*R², Bayesian coefficient of determination; SE, standard error; CrI, credible interval.*

**Supplementary File 19. Study-Level Posterior Estimates**

| **Study** | **Estimate** | **95% CrI** |
| --- | --- | --- |
| EdwinCChin2022 | −1.088 | [−1.470, −0.724] |
| AhmadAliAkbariKamrani2014 | −0.997 | [−1.480, −0.568] |
| XiaoyunZhou2026 | −0.980 | [−1.460, −0.544] |
| TsengHauTseng2020 | −0.963 | [−1.390, −0.561] |
| ShahrianEshaghi2019 | −0.898 | [−1.436, −0.405] |
| MeiFengTang2010 | −0.891 | [−1.290, −0.513] |
| MTadayon2016 | −0.863 | [−1.296, −0.446] |
| IulianaHartescu2015 | −0.822 | [−1.278, −0.388] |
| SeanGMcKenna2021 | −0.820 | [−1.310, −0.323] |
| SabaKarimi2016 | −0.795 | [−1.238, −0.369] |
| PaulineBaron2023 | −0.802 | [−1.194, −0.349] |
| DanSong2019 | −0.748 | [−1.160, −0.329] |
| DaniloTakashiAoike2017 | −0.716 | [−1.089, −0.356] |
| FeifeiWang2021 | −0.718 | [−1.154, −0.285] |
| DannyJYu2023 | −0.699 | [−1.119, −0.274] |
| HuiMeiChen2016 | −0.681 | [−1.067, −0.309] |
| SterianiElavsky2007 | −0.613 | [−1.042, −0.176] |
| AbbyCKing2002 | −0.553 | [−0.995, −0.104] |
| FeifeiWang2020 | −0.510 | [−0.977, −0.005] |
| TomasFritz2011 | −0.451 | [−0.849, −0.076] |
| JenniferAWenzel2013 | −0.444 | [−0.918, 0.026] |
| Pooled Effect | −0.763 | [−0.989, −0.548] |

*CrI, credible interval*
